# Visualized Experimental Investigation of Flow-Field Reconstruction and Enhanced Oil Recovery by Heterogeneous-Phase Composite Flooding in Complex Narrow-Channel Reservoirs

**DOI:** 10.3390/gels12080752

**Published:** 2026-08-21

**Authors:** Xianmin Zhang, Junzhi Yu, Kuiqian Ma, Lei Zhang, Yue Wang, Fei Shi

**Affiliations:** 1 State Key Laboratory of Deep Oil and Gas, China University of Petroleum (East China), Qingdao 266580, China; 2 Shengli Oil Production Plant, Shengli Oilfield, China Petrochemical Corporation, Dongying 257015, China; yujunzhi1031@126.com; 3 Bohai Petroleum Research Institute, Tianjin Branch of CNOOC (China) Ltd., Tianjin 300450, China; makq@cnooc.com.cn (K.M.); zhanglei13@cnooc.com.cn (L.Z.); wangyue10@cnooc.com.cn (Y.W.); shifei@cnooc.com.cn (F.S.)

**Keywords:** narrow-channel reservoirs, heterogeneous phase composite flooding, visualized physical modeling, remaining-oil remobilization, enhanced oil recovery

## Abstract

Complex narrow-channel reservoirs are strongly constrained by depositional architecture, resulting in highly nonuniform areal waterflood sweep, and pronounced water-cut variations among different channel types. These characteristics pose substantial challenges to stabilizing oil production and controlling water cut at high water-cut stages. To elucidate how narrow-channel planform architecture controls waterflood sweep, gel-assisted flow-field regulation by heterogeneous-phase composite flooding (HPCF), and remaining-oil mobilization, three representative configurations were reproduced in two-dimensional visual physical models. Sequential waterflood–HPCF–post-waterflood experiments were conducted, and time-lapse images and dynamic production data were integrated to characterize sweep evolution and remaining-oil mobilization across displacement stages. The results demonstrate that narrow-channel architecture exerts primary control on preferential flow-path development, gel migration and retention, spatial fluid redistribution, and displacement performance. During waterflooding, injected water preferentially migrated through high-permeability zones along channel centerlines, leaving channel margins, branch termini, and poorly connected regions insufficiently swept. After HPCF injection, the gel-containing composite system preferentially entered the established dominant flow paths. Gel retention and accumulation selectively increased flow resistance in these pathways, while mobility control induced subsequent fluids to divert toward bypassed regions, thereby enlarging the macroscopic swept volume and improving local displacement efficiency. A low injection rate promoted sustained gel-assisted flow diversion within bifurcated channels, whereas a high injection rate facilitated gel-slug propagation against the geometric constraints of highly sinuous channels and expanded its spatial coverage. Compared with waterflooding alone, HPCF increased the ultimate oil recovery of the three channel models by 19.23–26.47 percentage points. These findings clarify the coupled effects of narrow-channel architecture, gel transport and injection parameters on the profile-control and oil-recovery performance of HPCF, providing a mechanistic basis for water control and development optimization in high-water-cut narrow-channel reservoirs.

## 1. Introduction

Complex narrow-channel reservoirs are widespread in continental fluvial successions and offshore shallow-water clastic systems and represent important targets for enhanced oil recovery during late-stage waterflooding. Unlike laterally extensive amalgamated sand bodies, sheet sands, and broad channel belts, these reservoirs are characterized by narrow and geometrically complex sand bodies, rapid thickness variations, abrupt lateral facies transitions, variable branch connectivity, and pronounced internal heterogeneity [1,2]. Physical-modeling and streamline-based studies have shown that channel geometry, connectivity, and well-pattern placement jointly control waterflood sweep, remaining-oil distribution, and the effectiveness of development adjustments [3,4]. Waterflood behavior therefore reflects the interplay between pore-scale displacement properties—such as pore-throat structure, permeability contrast, and fluid mobility ratio—and reservoir-scale architecture, including channel boundaries, branching and amalgamation, curvature, and injection–production configuration. These controls redistribute pressure gradients and constrain hydraulic connectivity. Consequently, injected water tends to concentrate along high-permeability channel axes, branch-confluence zones, and other well-connected pathways rather than advancing as a uniform displacement front.

After prolonged waterflooding, preferential pathways established during early development become persistent, allowing injected water to circulate rapidly along low-resistance streamlines. Water-cut consequently increases, water-utilization efficiency declines, and incremental recovery diminishes [5]. Substantial volumes of oil nevertheless remain in channel margins, branch termini, curvature-shielded zones, local low-permeability compartments, and regions poorly connected to the dominant injection–production pathways [6]. This distribution cannot be attributed solely to pore-scale trapping; it also reflects the effects of channel geometry, connectivity, boundary confinement, and flow localization. The principal development challenge therefore shifts from supplementing displacement energy to reorganizing the established flow field and redirecting injected fluid toward bypassed regions. Increasing the injection rate alone may raise local pressure gradients but can also intensify short-circuiting through preferential pathways, yielding limited improvement in areal sweep [7,8].

Several strategies have been developed to mitigate inefficient water cycling and mobilize remaining oil at high water cut, including injection–production adjustment, well-pattern reconstruction, streamline optimization, cyclic water injection, deep conformance control, and chemical flooding [9,10]. Well-pattern and injection–production optimization primarily modify the pressure field and redirect streamlines, whereas chemical treatments alter flow resistance within preferential pathways; their coordinated application can improve access to bypassed oil [11,12]. Integrated approaches, such as multibranch relay chemical flooding, further emphasize the joint optimization of well configuration, flow pathways, and chemical systems [13]. However, in complex narrow-channel reservoirs, persistent preferential flow is strongly imposed by channel geometry and connectivity. Sustainable recovery improvement therefore requires direct regulation of these pathways in addition to pressure-field adjustment.

Chemical conformance control provides a means of achieving such regulation. Polymers improve mobility control, surfactants enhance microscopic displacement efficiency, and particulate agents such as preformed particle gels (PPGs) selectively increase flow resistance in high-permeability pathways through retention, deformation, plugging, and remigration [14,15,16,17]. The effectiveness of particle systems depends on their mechanical properties, particle–pore-throat compatibility, injection conditions, transport stability, and retention capacity [18,19,20,21,22,23,24]. Heterogeneous-phase composite flooding (HPCF) integrates these complementary functions: polymer-induced viscosity enhancement stabilizes displacement, particles divert flow away from preferential pathways, and surfactants mobilize capillary-trapped oil. Core-flooding, sandpack, two-dimensional physical-model, and visualization studies have shown that HPCF performance is jointly governed by formulation, reservoir heterogeneity, slug design, injection strategy, and subsequent waterflooding [25,26]. Combining hydrolyzed polyacrylamide with branched PPGs improves particle retention and flow diversion after polymer flooding [27,28], while analogous synergy has been reported for PPG-enhanced alkali–surfactant–polymer systems [29]. Slug configuration and sustained particle migration further control system placement and sweep expansion [30,31]. Online saturation monitoring and visualization experiments have linked these transport processes to flow redistribution and remaining-oil mobilization across pore and model scales [32,33,34,35,36].

Despite this progress, the mechanisms by which HPCF reorganizes flow in complex narrow-channel reservoirs remain insufficiently resolved. Three limitations are particularly important. First, most previous studies have relied on one-dimensional cores, sandpacks, or idealized heterogeneous models. Although these approaches quantify injectivity, plugging performance, and incremental recovery, they do not adequately represent the effects of confined boundaries, branch connectivity, and channel curvature on areal flow evolution. Second, performance has generally been evaluated using bulk responses, including pressure, water cut, recovery factor, and agent retention. Direct visual evidence remains limited regarding HPCF propagation through complex channel geometries, its interception of established preferential pathways, the resulting flow diversion, and the spatial redistribution of remaining oil. Third, channel configuration, injection rate, and well-pattern deployment exert coupled controls on displacement, but their combined effects on HPCF-induced flow-field reorganization and remaining-oil mobilization have not been systematically quantified.

In this study, channel configuration refers specifically to the two-dimensional planform geometry represented by the physical models, including channel morphology, branch connectivity, intersection and amalgamation, sinuosity, and lateral boundaries. It does not encompass the complete three-dimensional reservoir architecture. Within this defined scope, the study addresses three questions: how preferential water-flow pathways develop under different channel configurations; how HPCF interacts with these pathways to induce flow diversion; and how channel configuration, injection rate, and well-pattern deployment jointly control the remobilization of remaining oil.

To answer these questions, three representative planform configurations—an upstream section of a bifurcated channel, a midstream section of a bifurcated channel, and a high-sinuosity channel—were abstracted and incorporated into two-dimensional visual heterogeneous physical models. Sequential waterflooding–HPCF–post-HPCF waterflooding experiments were conducted under different injection rates and well-pattern configurations. Time-lapse image analysis was integrated with production dynamics, recovery factor, sweep efficiency, and displacement efficiency to resolve preferential-pathway development during initial waterflooding, selective resistance build-up and flow diversion during HPCF injection, and remaining-oil mobilization during subsequent waterflooding. This stage-resolved approach links HPCF migration directly to the spatiotemporal evolution of the displacement field, thereby clarifying the coupled mechanisms of preferential-pathway regulation, flow diversion, and remaining-oil mobilization under geometrically confined conditions. The findings provide a mechanistic experimental basis for optimizing HPCF application in complex narrow-channel reservoirs at high water cut.

## 2. Results and Discussion

### 2.1. Displacement Experiment in the Front-Section of a Bifurcated Channel

For the front-section of a bifurcated channel, where pronounced flow-field heterogeneity promotes preferential transport along individual branches, four displacement experiments were conducted using a 2D visual physical model, with injection rate and well-pattern configuration as the principal control variables. The experiments were designed to characterize the macroscopic retention and plugging behavior of HPCF, the associated streamline reconfiguration, and the synergistic mobilization of bypassed oil in interbranch regions and remaining oil in retention zones. The experimental schemes are as follows:

Scheme 1—Basic waterflooding (3 mL·min^−1^): This scheme served as the reference case for characterizing the development of preferential flow paths and the remaining oil distribution under waterflooding conditions.

Scheme 2—High-rate waterflooding (4 mL·min^−1^): Comparison with Scheme 1 was used to investigate the effects of increased injection rate on areal sweep, and displacement performance during waterflooding.

Scheme 3—Waterflooding + HPCF divergent profile control and flooding: A one-injector-three-producer (1I-3P) well pattern was employed at 3 mL·min^−1^ to evaluate the plugging and fluid-diversion performance of HPCF under a divergent flow field.

Scheme 4—Waterflooding + HPCF convergent profile control and flooding: A three-injector-one-producer (3I-1P) well pattern was employed at 3 mL·min^−1^. Comparison with Scheme 3 enabled evaluation of HPCF-induced mobilization of remaining oil in strongly stagnant regions and enhancement of deep fluid diversion under multidirectional convergent-flow conditions.

#### 2.1.1. Regulation Experiments at Different Injection Rates

Waterflooding in narrow-channel reservoirs is strongly constrained by channel geometry and depositional heterogeneity. The channel axis generally exhibits higher permeability and better pore connectivity than the margins, directing injected water preferentially through the channel center. This axis-focused flow promotes early breakthrough while leaving lower-permeability marginal zones poorly swept. Continued injection further stabilizes the central low-resistance pathway, concentrates the streamlines, and intensifies water channeling, thereby progressively reducing areal sweep efficiency.

**Figure 1:**
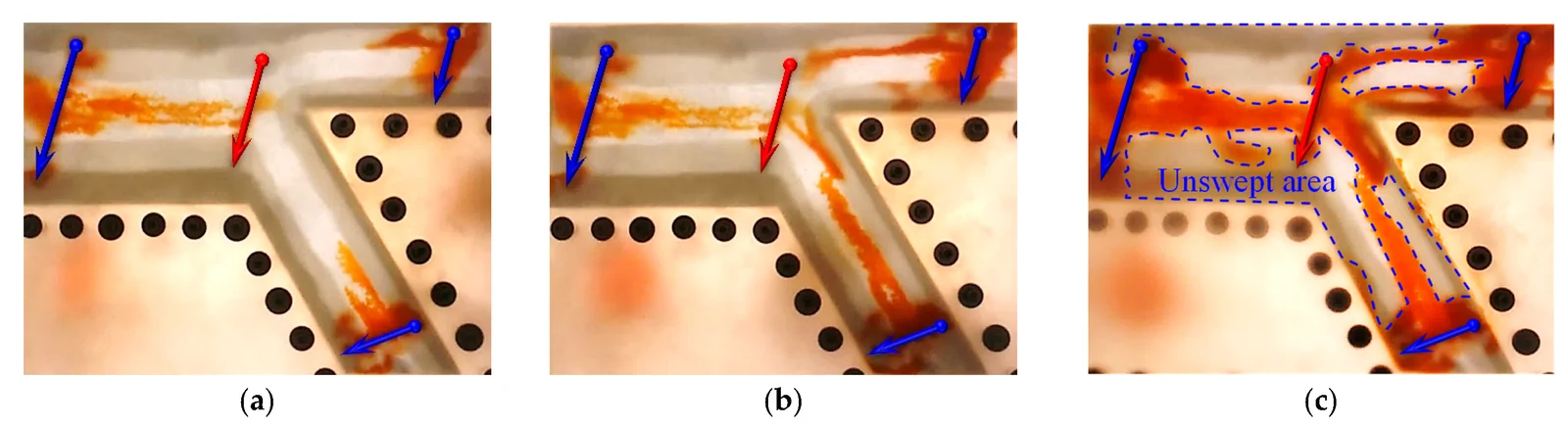
Visual results of waterflooding in the front-section model of a bifurcated channel at 3 mL·min^−1^: (**a**) initial stage; (**b**) waterflood front breakthrough; (**c**) end of waterflooding.

**Figure 2:**
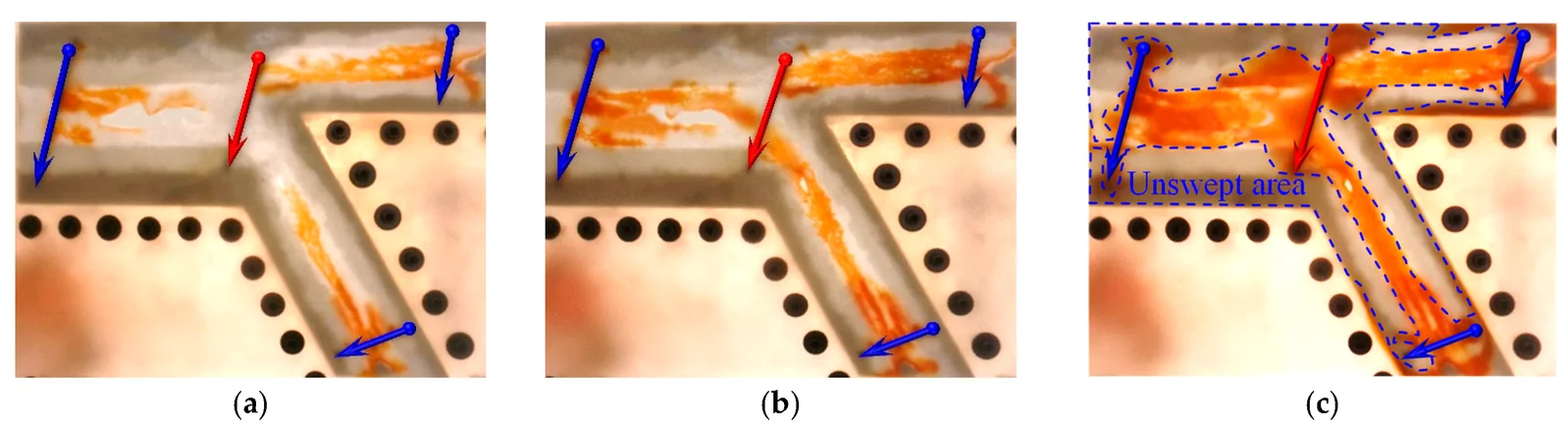
Visual results of waterflooding in the front-section model of a bifurcated channel at 4 mL·min^−1^: (**a**) initial stage; (**b**) waterflood front breakthrough; (**c**) end of waterflooding.

As shown in Figure 1 and Figure 2, injection rate primarily controls the rate of front propagation and the synchronization of displacement among connected channel segments. At the lower injection rate, the limited pressure gradient produces slow front advancement and amplifies the response differences associated with variations in channel width and connectivity. Water first breaks through along the low-resistance main channel, whereas branch channels and marginal zones respond more slowly, resulting in strongly asynchronous displacement. Increasing the injection rate raises the viscous pressure gradient, accelerates front propagation, and reduces the breakthrough lag between connected channel segments. However, it does not fundamentally reorganize the dominant flow pathways. Under both injection conditions, streamlines remain concentrated along the high-permeability channel axis, lateral invasion into lower-permeability margins is limited, and substantial marginal oil remains bypassed. Injection rate therefore governs displacement kinetics, whereas channel architecture controls the spatial pattern and ultimate extent of sweep.

**Figure 3:**
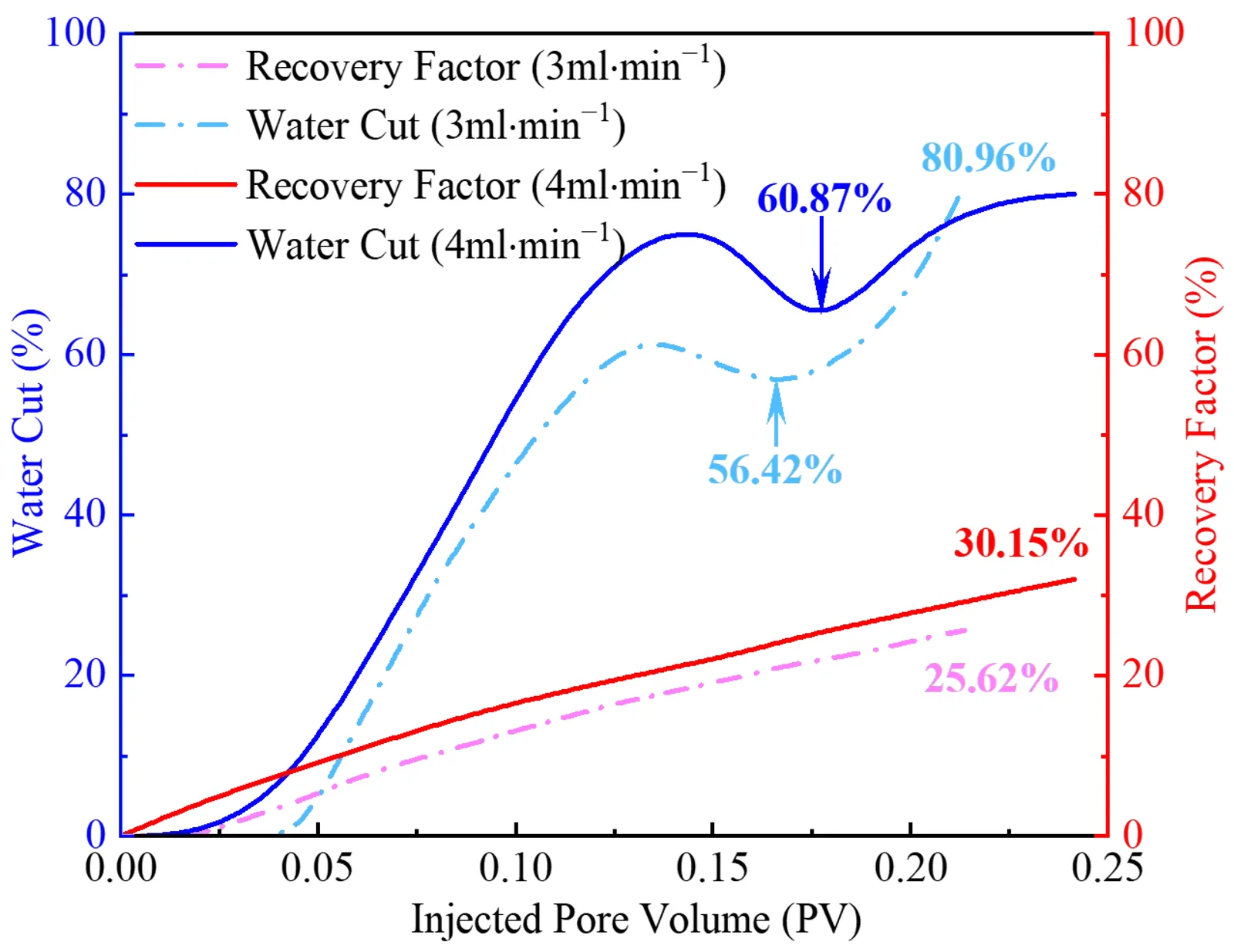
Recovery and water cut curves for the front-section model of a bifurcated channel at different injection rates.

Injection rate significantly influenced waterflood performance and water-cut response (Figure 3). Increasing the injection rate raised the final recovery factor from 25.62% to 30.15%, corresponding to an increase of 4.53 percentage points. Although high-rate injection shortened the water-free production period, it produced a higher oil-production rate before water breakthrough, indicating more rapid pressure communication and early oil mobilization. This trade-off may be advantageous for offshore developments that prioritize short production cycles and high operational efficiency, provided that the associated increase in water-handling demand remains manageable.

Under both injection conditions, the water-cut curves fluctuated during the middle- to high-water-cut stages, typically showing a transient decline followed by a rapid rebound. The larger fluctuations observed at the higher injection rate indicate that stronger injection intensified front instability and spatially nonuniform displacement. These fluctuations resulted from the combined effects of reservoir heterogeneity and asynchronous front propagation. Water first broke through along the high-permeability main channel, causing the initial rise in water cut, whereas delayed displacement in branch channels and lower-permeability regions generated subsequent transient variations. Differences in flow-path length and hydraulic resistance between individual injectors and the producer further caused the displacement fronts to arrive at different times. The resulting superposition of production streams from regions at different displacement stages amplified the observed water-cut fluctuations.

#### 2.1.2. Regulation Experiments Under Different Well-Pattern Configurations

**Figure 4:**
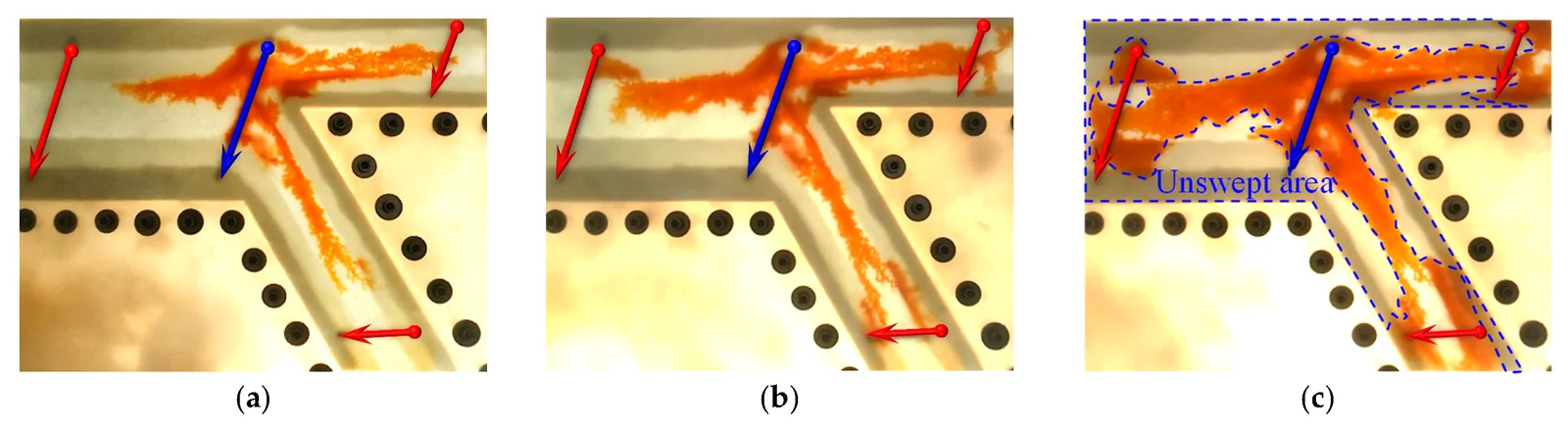
Visual results of waterflooding the front-section model of a bifurcated channel under the 1I–3P configuration: (**a**) initial stage, (**b**) waterflood-front breakthrough, and (**c**) end of waterflooding.

**Figure 5:**
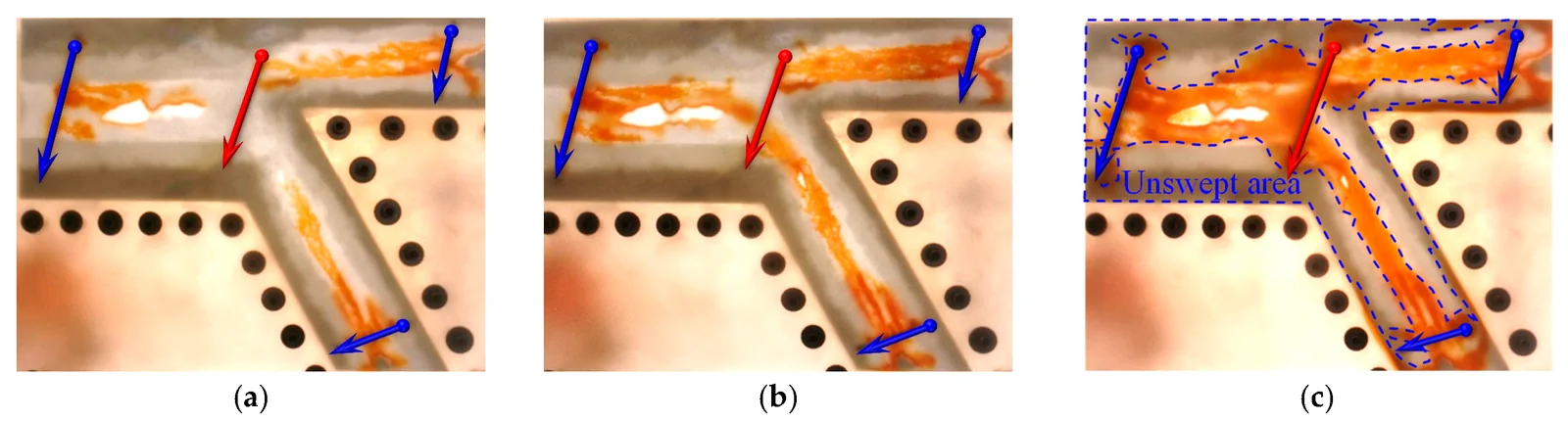
Visual results of waterflooding in the front-section model of a bifurcated channel under the 3I–1P configuration: (**a**) initial stage, (**b**) waterflood-front breakthrough, and (**c**) end of waterflooding.

Figure 4 and Figure 5 demonstrate that well-pattern configuration strongly controls flow-field evolution and waterflood sweep in the front-section model of a bifurcated channel. Under the one-injector–three-producer (1I–3P) configuration, injected water diverges from a single injector toward multiple producers. The displacement front preferentially propagates along the high-permeability channel axis, while lateral channel boundaries restrict transverse fluid expansion and further concentrate the streamlines toward the central low-resistance pathway. This behavior promotes early breakthrough and severe water channeling, producing rapid axial advancement but limited sweep of the channel flanks. Consequently, remaining oil accumulates primarily along the margins and in zones of relatively poor reservoir quality.

By contrast, the three-injector–one-producer (3I–1P) configuration transforms the flow field from divergent to multidirectional convergent flow. The interaction of fluids from multiple injectors generates overlapping sweep paths within the channel, enhancing fluid invasion into branch channels and marginal zones. This configuration partially suppresses preferential breakthrough along the central high-permeability pathway, expands the overall swept area, and improves the uniformity of areal oil mobilization.

During injection, the slug accumulates within the central high-permeability zone, where B-PPG gel particles progressively increase the hydraulic resistance through migration, elastic deformation, retention, and local accumulation. This gel-mediated resistance weakens the established preferential flow pathway and promotes the diversion of subsequently injected water from the main channel toward low-permeability margins and previously unswept regions, thereby enhancing the mobilization of marginal remaining oil.

**Figure 6:**
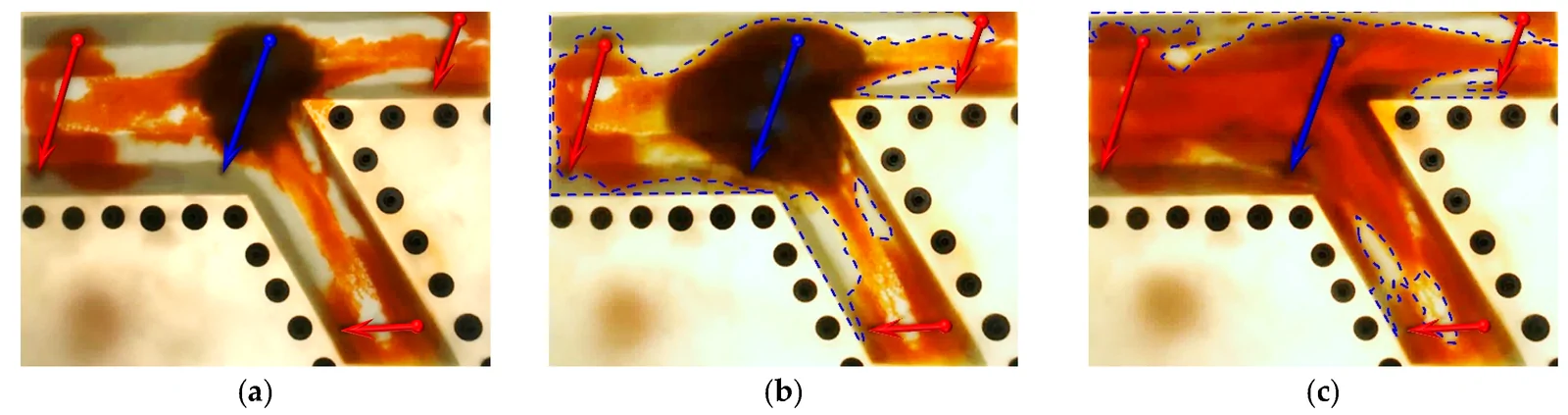
Visual results of HPCF displacement in the front-section model of a bifurcated channel under the 1I–3P configuration: (**a**) onset of HPCF injection, (**b**) end of HPCF injection, and (**c**) end of subsequent waterflooding.

**Figure 7:**
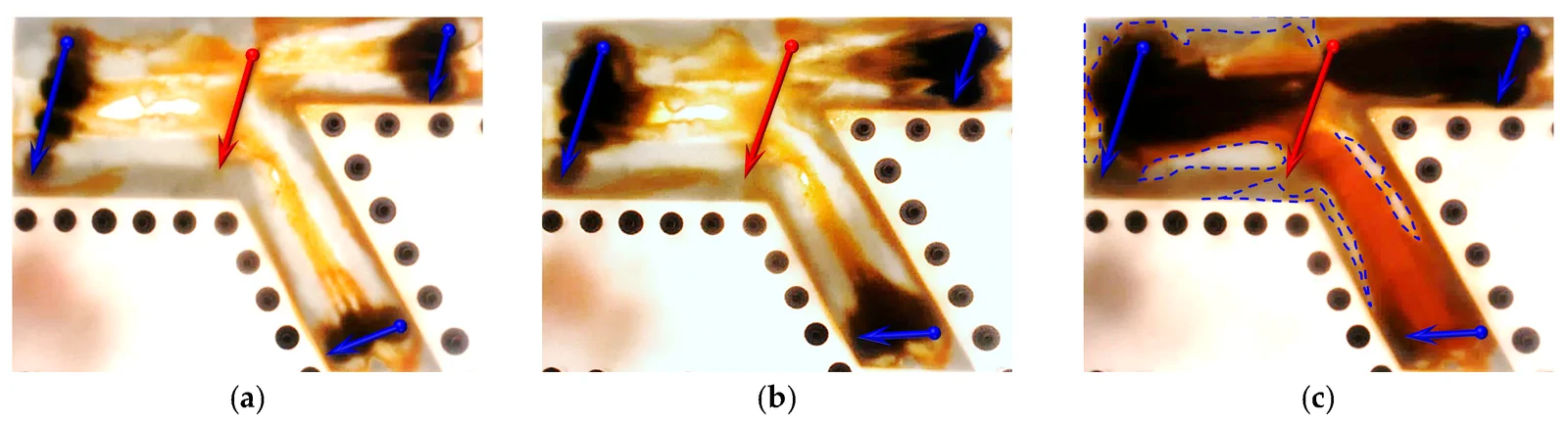
Visual results of HPCF displacement in the front-section model of a bifurcated channel under the 3I–1P configuration: (**a**) onset of HPCF injection, (**b**) end of HPCF injection, and (**c**) end of subsequent waterflooding.

Comparison of the two well patterns reveals that the 3I–1P configuration has distinct limitations in flow-field reconfiguration during HPCF injection (Figure 6 and Figure 7). Although multidirectional convergent flow improves areal sweep during waterflooding, simultaneous HPCF injection from three spatially separated injectors disperses both the driving force and slug distribution, resulting in more dispersed migration and retention of B-PPG gel particles along different preferential flow pathways. Consequently, sustained retention and local accumulation of B-PPG particles are insufficient to establish a strong and continuous high-resistance zone near the main-channel confluence, thereby weakening sustained control of preferential water pathways. Moreover, the distributed injection pattern generates an insufficient transverse pressure gradient between the dominant pathway and poorly swept margins, limiting deep fluid diversion and further remaining-oil mobilization.

By contrast, under the 1I–3P configuration, concentrated HPCF injection from a single inlet rapidly establishes a continuous resistance zone within the high-permeability main channel. This configuration more effectively suppresses the preferential pathway and redirects subsequent water toward the channel margins. The 1I–3P pattern is therefore more favorable for preferential-pathway control, lateral streamline diversion, and marginal remaining-oil mobilization during HPCF treatment.

**Figure 8:**
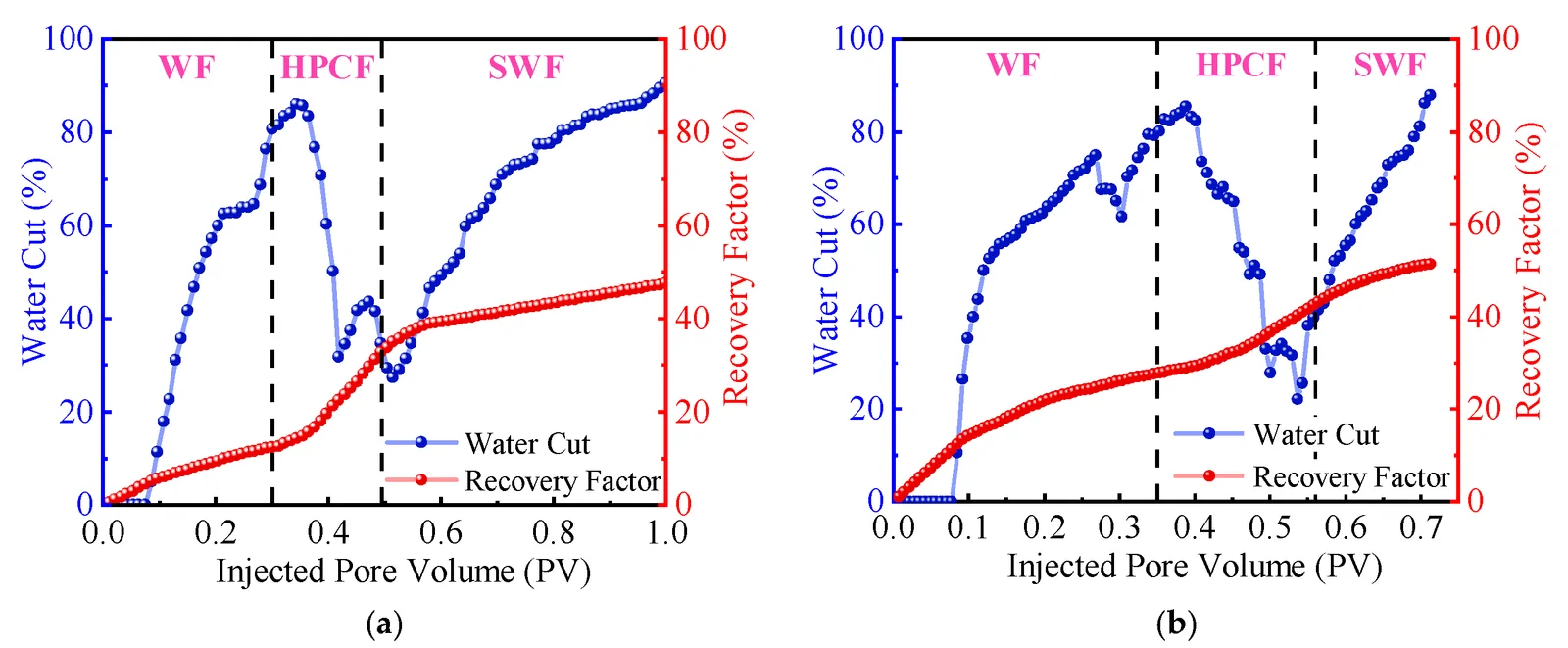
Water-cut and recovery-factor curves for the front-section model of a bifurcated channel under different well-pattern configurations: (**a**) 1I–3P and (**b**) 3I–1P.

During waterflooding, the 1I–3P configuration exhibited a relatively short water-free production period, followed by a rapid, convex increase in water cut after breakthrough (Figure 8). Water preferentially propagated from the single injector along the high-permeability main channel, resulting in early establishment of a dominant flow pathway. Continued injection reinforced this low-resistance pathway, causing the water cut to rise rapidly while the recovery-factor increment progressively diminished. By contrast, the 3I–1P configuration prolonged the water-free production period and produced a stagewise water-cut response, including a local oscillation and decline near 0.25 PV. This behavior reflects asynchronous front propagation through individual branches and dynamic interference among multidirectional inflows near the producer. At later stages, successive breakthrough of multiple branches established a new preferential-flow pattern, leading to a renewed rapid increase in water cut.

**Figure 9:**
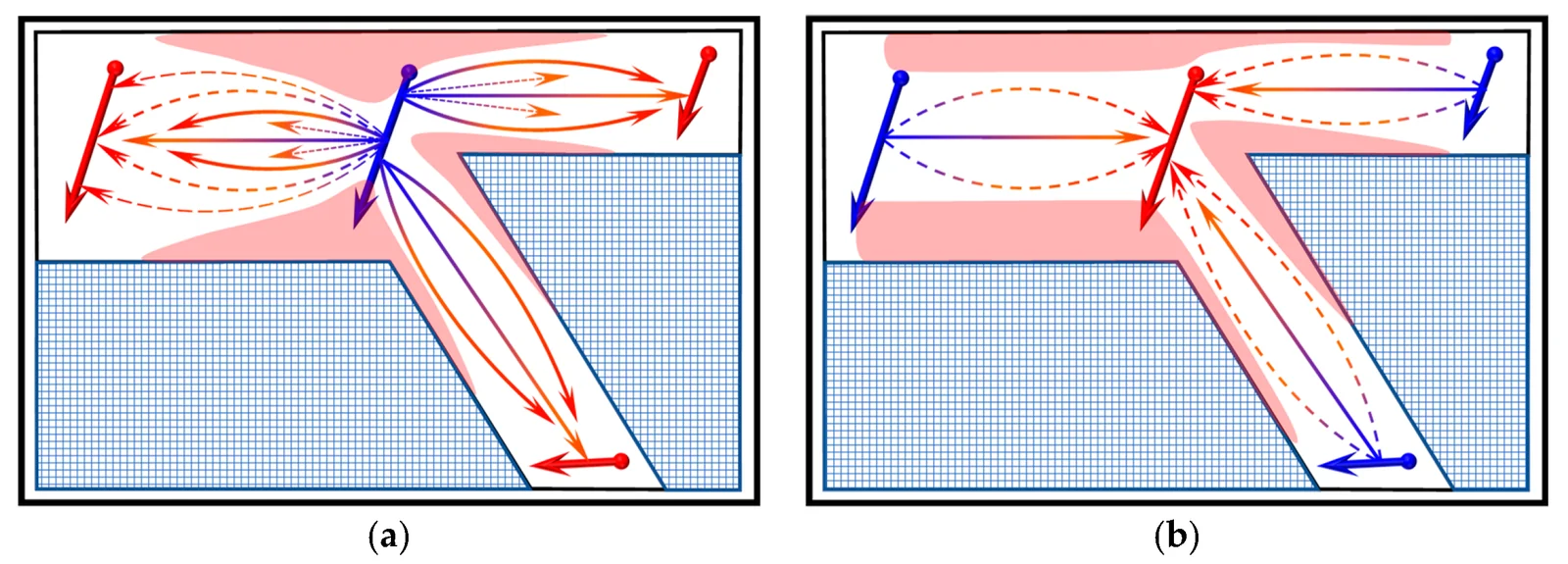
Schematic streamline distributions in the front-section model of a bifurcated channel under different well-pattern configurations: (**a**) 1I–3P and (**b**) 3I–1P.

Following HPCF injection, both well patterns exhibited a pronounced decrease in water cut and an increased recovery-factor slope, indicating that the original high-water-cut flow field was significantly reconfigured under the regulation of B-PPG (Figure 9). Under the 1I–3P configuration, concentrated injection generated a relatively continuous slug distribution along the dominant pathway, producing a more persistent improvement in the sweep of branch channels and marginal zones. Under the 3I–1P configuration, convergence of the injected slugs produced strong localized resistance enhancement, resulting in a steeper initial water-cut decline and faster short-term recovery response. However, because the HPCF slug was distributed among multiple injectors, this effect was less persistent and primarily reflected localized intensification rather than spatially balanced flow regulation.

**Figure 10:**
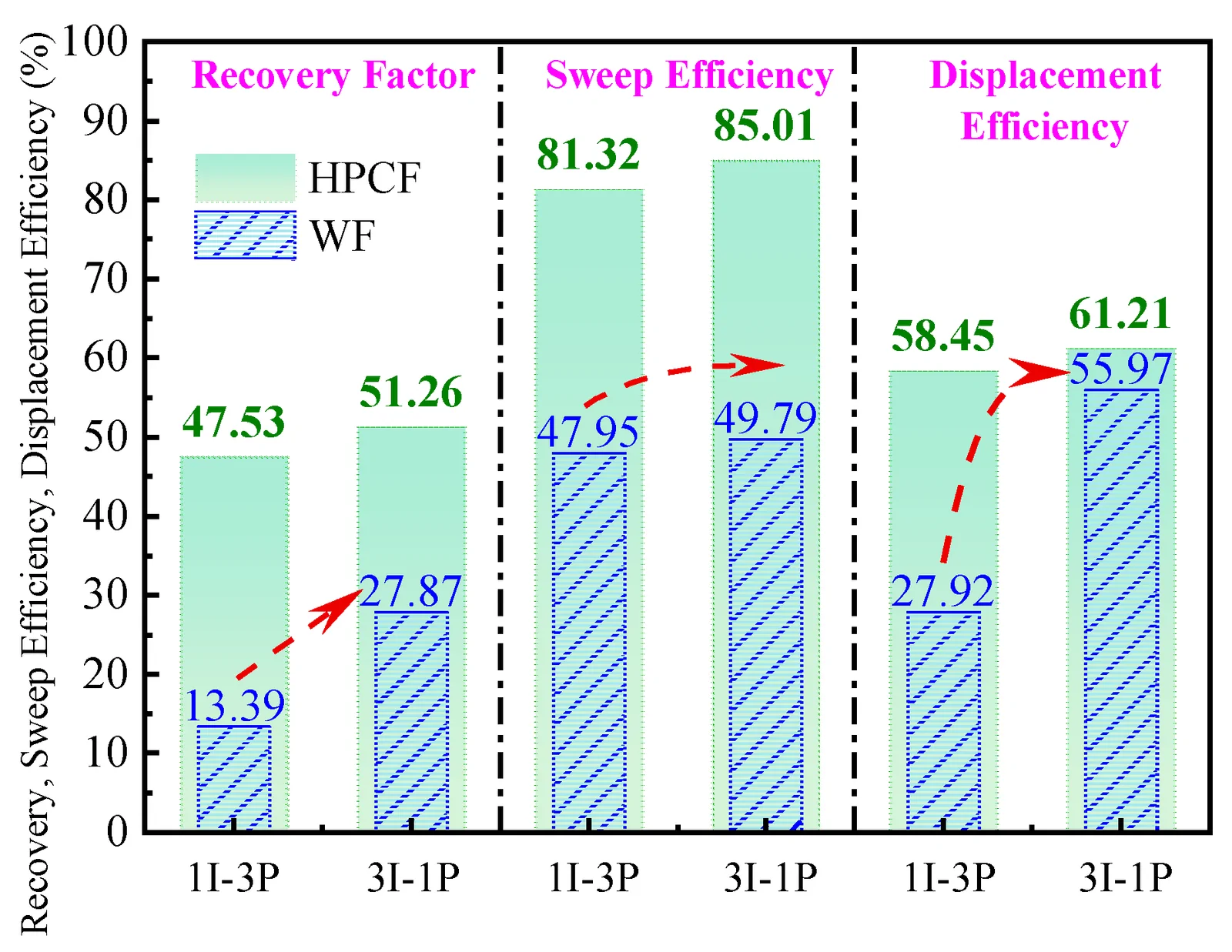
Comparison of recovery factor, sweep efficiency, and displacement efficiency after waterflooding and HPCF injection under different well-pattern configurations in the front-section model of a bifurcated channel.

Well-pattern configurations exhibited distinct stage-dependent advantages (Figure 10). During waterflooding, the 3I–1P configuration increased the recovery factor from 13.39% to 27.87% relative to the 1I–3P configuration. This improvement arose primarily from more uniform displacement and higher displacement efficiency rather than substantial expansion of the swept area. Under the 1I–3P configuration, contrasts in channel transmissibility concentrated the injected water within the high-permeability main channel, limiting fluid allocation to the branches and intensifying areal sweep heterogeneity. In contrast, multipoint injection under the 3I–1P configuration redistributed flow among the channel branches and suppressed breakthrough dominated by a single preferential pathway, thereby improving interbranch displacement uniformity and waterflood recovery.

The incremental-recovery mechanisms differed markedly during HPCF injection. Under the 3I–1P configuration, the relatively high degree of reservoir contact achieved during waterflooding left less mobilizable oil. HPCF therefore produced only a limited increase in displacement efficiency, which reached 61.21%; its primary contribution was the expansion of the swept area through preferential-pathway plugging and streamline redistribution, increasing the sweep coefficient to 85.01%. By contrast, the lower waterflood mobilization under the 1I–3P configuration preserved greater remaining-oil potential. HPCF both expanded the swept area, increasing the sweep coefficient to 81.32%, and improved mobility control and local displacement conditions, raising the displacement efficiency to 58.45%. Thus, for well patterns with poor initial waterflood performance, HPCF can simultaneously improve sweep and displacement efficiencies, yielding a stronger overall incremental-recovery response.

**Figure 11:**
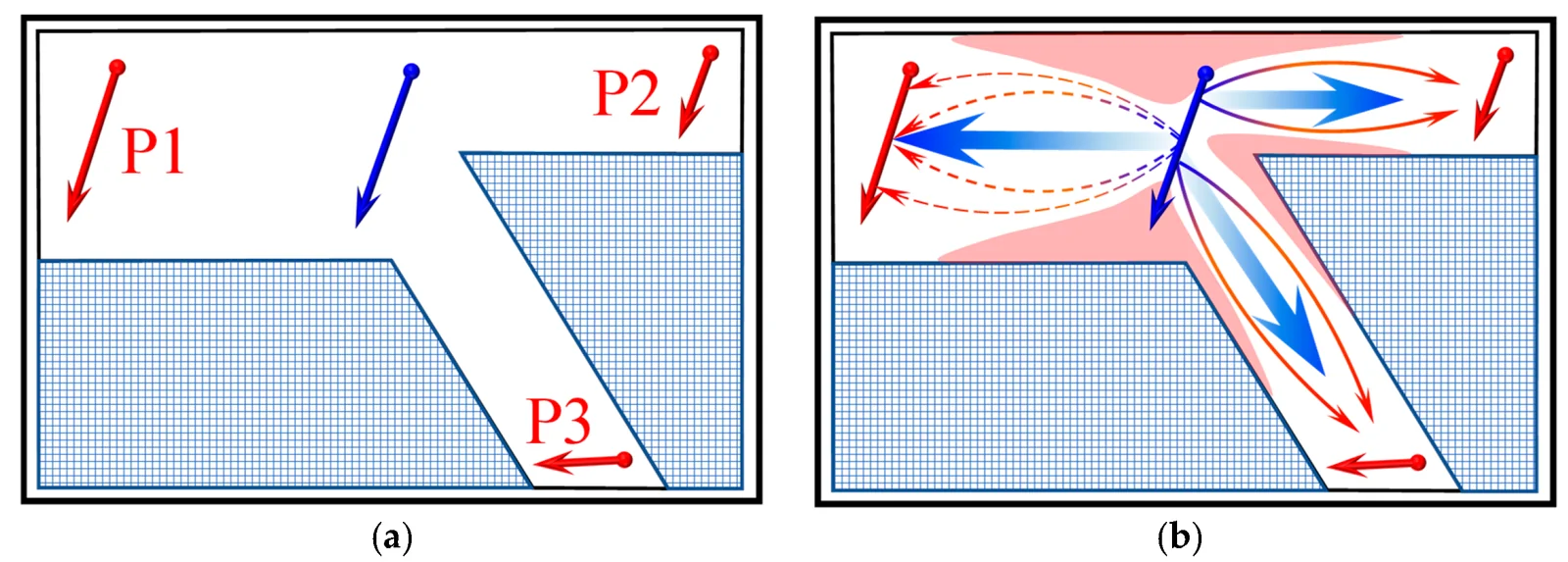
Schematic diagram of well placement and streamline response under the 1I–3P configuration: (**a**) well placement; (**b**) time-dependent streamline response.

**Figure 12:**
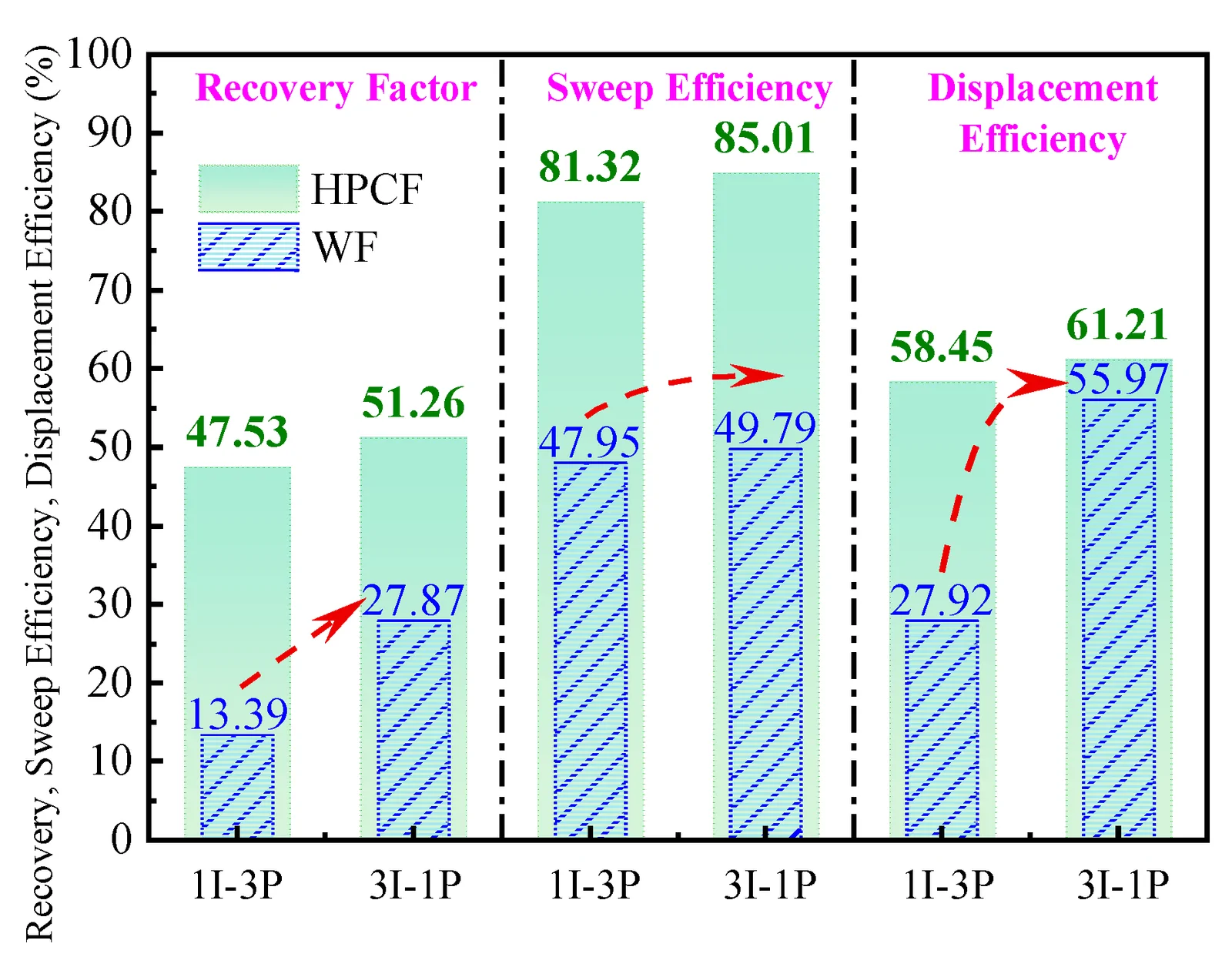
Water-cut curves of individual production wells under the 1I–3P configuration in the front-section model of a bifurcated channel.

To elucidate the position-dependent displacement behavior under the one-injector/three-producer well pattern, the development responses of the P1-controlled main-channel region and the P2- and P3-controlled branch-channel regions were compared (Figure 11 and Figure 12). The results show that variations in channel width, hydraulic connectivity, and local petrophysical properties govern the spatial heterogeneity of displacement performance. During waterflooding, the main-channel region controlled by well P1 exhibits stronger connectivity and lower flow resistance, facilitating relatively uniform water advance, a rapid increase in water cut, and a high initial degree of oil mobilization. In contrast, the P2- and P3-controlled regions are located within branch channels, where areal heterogeneity and localized low-permeability zones induce nonuniform water advance, delay the water-cut response, and restrict areal sweep. Especially in the P3-controlled region, injected water has difficulty effectively entering the low-permeability area, resulting in a relatively large range of unswept zones and remaining-oil enrichment areas. As development progresses, the P1-controlled region sustains a relatively high mobilization level, whereas production growth in the P2- and P3-controlled regions gradually decelerates, with increasingly pronounced retention of remaining oil.

From the perspective of breakthrough characteristics, the breakthrough time in the P1 well region is relatively early, indicating that an effective flow channel is preferentially formed in the main channel. Breakthrough in the P2- and P3-controlled regions is relatively delayed, reflecting the slower establishment of displacement channels and insufficient mobilization degree in the branch channels. The differences in breakthrough time among different well regions reflect the imbalance in displacement intensity and flow allocation between the main channel and branch channels. As local preferential flow paths in the branch channels gradually form, the flow-field allocation relationship is adjusted, the displacement energy in some areas of the main channel weakens, and the displacement-front advancement slows. This indicates that the formation of preferential channels does not significantly expand the overall sweep range but is more manifested as the strengthening and fixation of preferential flow paths within existing channels.

After entering the HPCF stage, the response differences among the different well-controlled regions became more pronounced. In the P1-controlled region, the water-cut reduction was relatively limited and was followed by a rapid rebound, indicating that the main channel had already been extensively mobilized during the preceding waterflooding stage. After HPCF injection, the strongly developed preferential pathway in this region made it difficult for B-PPG gel particles to establish sustained and concentrated retention and resistance enhancement, thereby limiting further flow regulation and incremental oil recovery. In contrast, the P2- and P3-controlled regions are located within relatively narrow branch channels, where B-PPG particles are more prone to retention and local accumulation along preferential flow paths, increasing flow resistance and inducing subsequent fluid diversion toward insufficiently swept regions. During subsequent waterflooding, this redistribution improves fluid allocation and promotes further advancement of the displacement front into previously unswept areas, thereby expanding the swept region and enhancing remaining-oil mobilization within the branch channels.

Further, combined with the characteristics of water-cut variation, although the P1-controlled region shows an obvious water-cut reduction and oil-increase response during the heterogeneous flooding stage, the minimum water cut remains at a relatively high level. This indicates that although profile control and flooding effectively improve the mobilization condition of the main channel at this stage, its oil-increase potential is limited by the development of preferential channels in the early stage and the dispersed enrichment of remaining oil. Overall, under the 1I-3P configuration, the waterflooding stage is mainly characterized by preferential mobilization of the main channel and insufficient mobilization of branch channels, whereas the main function of the heterogeneous flooding stage is to provide supplementary regulation of insufficiently mobilized areas in branch channels, rather than to achieve another substantial potential exploitation of the highly water-washed areas in the main channel.

### 2.2. Displacement Experiment in the Middle Section of a Bifurcated Channel

#### 2.2.1. Visualized Displacement Behavior

**Figure 13:**
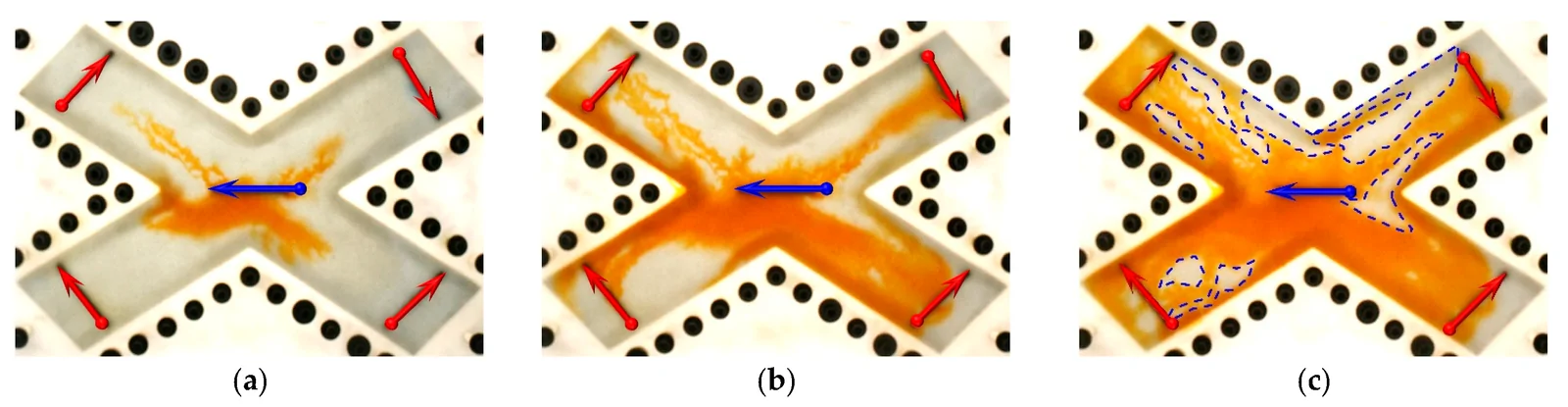
Visual results of waterflooding in the middle-section model of a bifurcated channel at 3 mL·min^−1^: (**a**) initial stage; (**b**) waterflood front breakthrough; (**c**) end of waterflooding.

**Figure 14:**
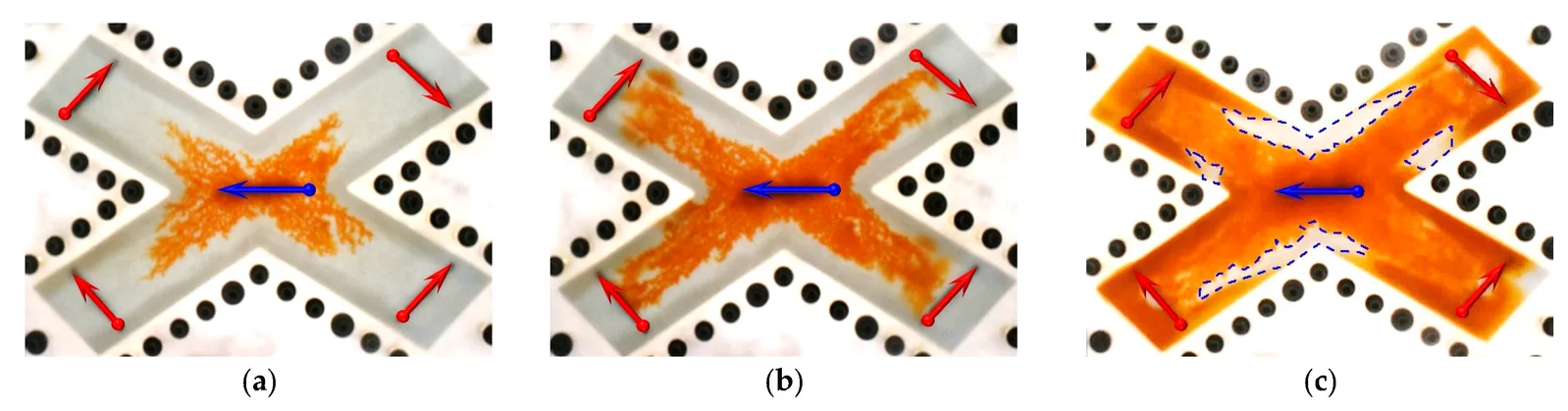
Visual results of waterflooding in the middle-section model of a bifurcated channel at 4 mL·min^−1^: (**a**) initial stage; (**b**) waterflood front breakthrough; (**c**) end of waterflooding.

As shown in Figure 13 and Figure 14, front evolution in the midstream bifurcated-channel model is jointly controlled by permeability heterogeneity and interbranch connectivity. Injected water preferentially follows the lowest-resistance pathway between the injector and producer, whereas injection rate primarily modulates the propagation rate and lateral extent of the displacement front.

At 3 mL·min^−1^, the limited pressure gradient and preferential flow through the high-permeability main channel generate a narrow, elongated front and strongly directional breakthrough. The branch flanks remain poorly contacted, resulting in dispersed patches of bypassed oil. At 4 mL·min^−1^, the higher pressure gradient promotes lateral invasion, broadens the front, and improves sweep within the main channel and adjacent branch regions. Accordingly, low-rate waterflooding leaves multiple oil-rich zones along the main channel, at branch termini, and near channel margins, whereas high-rate injection confines most remaining oil to lower-permeability and locally bypassed regions. Increasing the injection rate therefore improves oil mobilization in the midstream bifurcation zone mainly by expanding macroscopic sweep; it reduces, but does not eliminate, the preferential flow and displacement nonuniformity imposed by the channel architecture.

**Figure 15:**
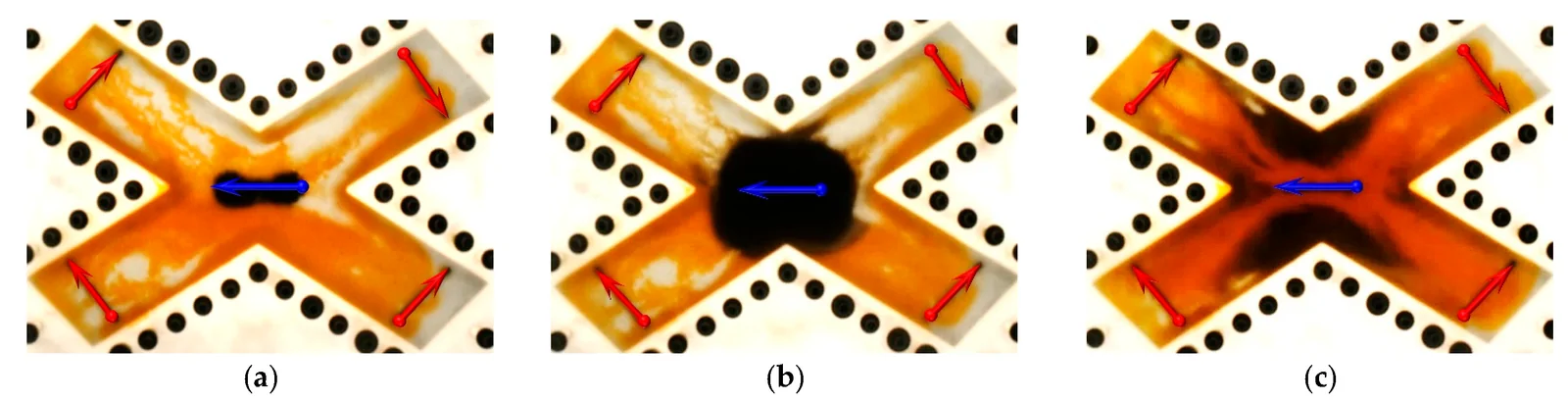
Visual results of HPCF in the middle-section model of a bifurcated channel at 3 mL·min^−1^: (**a**) initial injection stage; (**b**) end of heterogeneous-phase composite flooding; (**c**) end of subsequent waterflooding.

**Figure 16:**
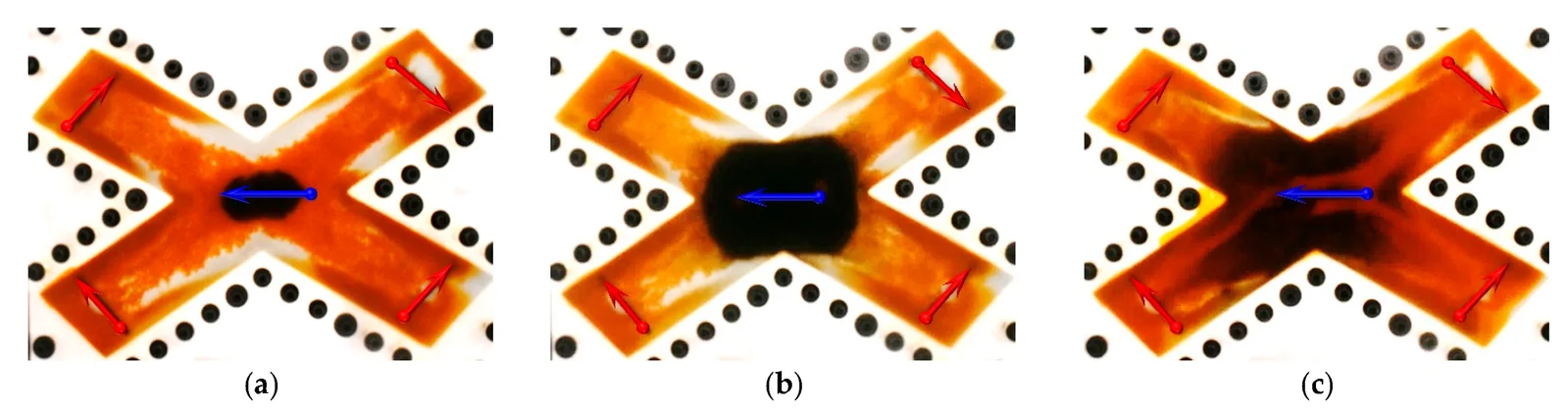
Visual results of HPCF in the middle-section model of a bifurcated channel at 4 mL·min^−1^: (**a**) initial injection stage; (**b**) end of heterogeneous-phase composite flooding; (**c**) end of subsequent waterflooding.

As shown in Figure 15 and Figure 16, HPCF performance in the middle-section model of a bifurcated channel is strongly dependent on injection rate, reflecting a trade-off between sustained flow regulation and displacement intensity. At 3 mL·min^−1^, the HPCF slug migrates relatively slowly and remains in contact with the high-permeability preferential pathway for a longer period. The prolonged residence time facilitates the retention and local accumulation of B-PPG gel particles, promoting the development of a relatively continuous high-resistance zone and suppressing flow through the original low-resistance pathway. As a result, subsequently injected fluids are diverted toward medium- and low-permeability regions, leading to a more uniform displacement front and sustained mobilization of previously bypassed oil.

At 4 mL·min^−1^, the greater driving force accelerates the propagation of the HPCF slug and the transport of B-PPG gel particles, thereby enhancing the overall displacement intensity and final oil mobilization. However, the faster transport shortens the residence time of B-PPG within the preferential pathway, reducing sustained particle retention and local accumulation and thus limiting the formation of a stable and continuous high-resistance zone. Consequently, subsequently injected fluids can re-establish breakthrough through locally weak-resistance regions, resulting in less persistent flow-field regulation than that observed at the lower injection rate. Therefore, low-rate injection is more favorable for B-PPG retention, selective resistance enhancement, and sustained streamline diversion, whereas high-rate injection promotes stronger displacement and greater final oil mobilization.

#### 2.2.2. Comparison of Displacement Performance

**Figure 17:**
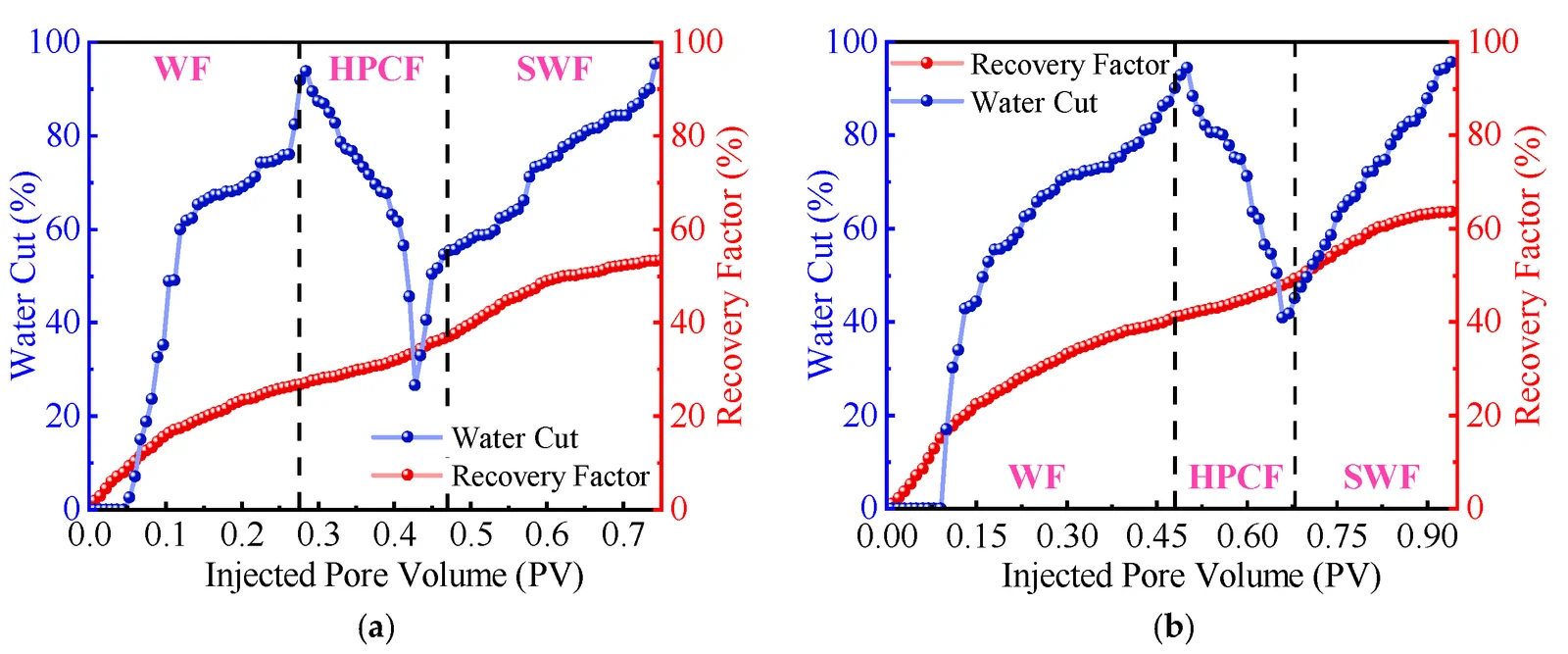
Water-cut and recovery-factor curves at different injection rates in the middle-section model of a bifurcated channel: (**a**) 3 mL·min^−1^; (**b**) 4 mL·min^−1^.

Figure 17 shows distinct stagewise responses corresponding to initial waterflooding, HPCF injection, and subsequent waterflooding. During initial waterflooding, both injection-rate cases exhibit a short water-free production period followed by a rapid increase in water cut, indicating establishment of hydraulic communication between the injector and producer. The water-cut curves display an overall convex shape, with rapid early growth followed by a slower increase and superimposed stepwise changes. This behavior reflects differences in interbranch connectivity and the asynchronous breakthrough of displacement fronts along multiple preferential pathways. As these pathways become established, the water-cut increase and recovery-factor growth gradually decelerate. At 3 mL·min^−1^, the curve slopes change relatively smoothly, indicating stable front propagation. By contrast, the more pronounced slope variations at 4 mL·min^−1^ reflect stronger local breakthrough and greater displacement nonuniformity. Thus, although the higher injection rate enhances the overall driving force, it also intensifies front instability during the principal advancement stage.

HPCF injection causes an immediate water-cut decline and a simultaneous increase in the recovery-factor slope, demonstrating effective disruption of the established high-water-cut flow regime. At 3 mL·min^−1^, the water cut decreases from 91.88% to 26.66%, with a reduction of 65.22 percentage points, while the recovery factor increases by 26.47 percentage points. At 4 mL·min^−1^, the water cut decreases from 90.18% to 40.85%, and the recovery factor increases by 23.26 percentage points. The stronger response at the lower rate confirms that longer slug residence enhances HPCF-induced flow regulation. Nevertheless, the higher-rate case achieves a greater final cumulative recovery because of its more effective initial waterflood mobilization. During subsequent waterflooding, the water cut in both cases gradually rebounds to approximately 95%, indicating attenuation of the HPCF effect. These results highlight a rate-dependent trade-off between slug-regulation efficiency and overall reservoir mobilization.

**Figure 18:**
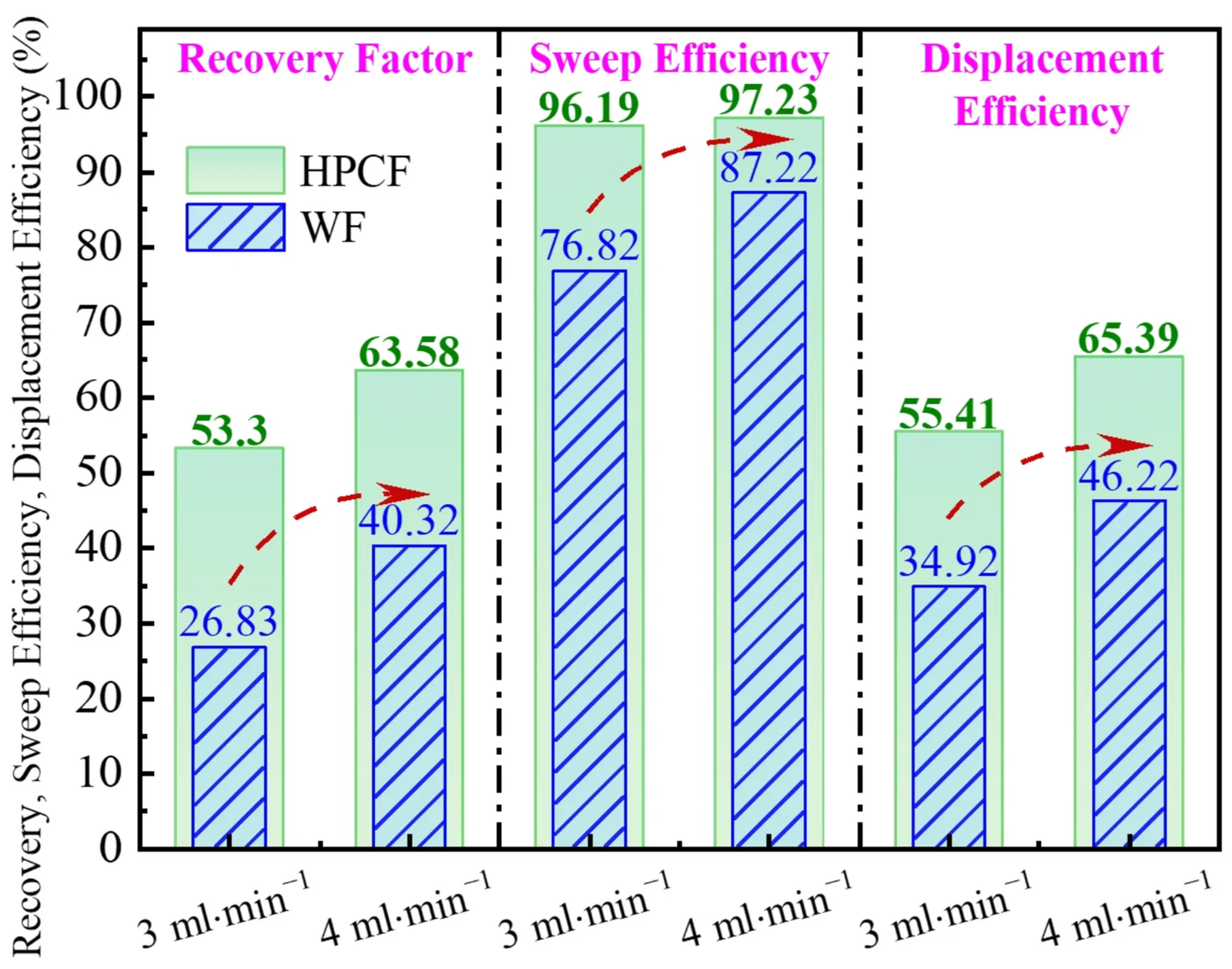
Comparison of recovery factor, sweep efficiency, and displacement efficiency after waterflooding and HPCF injection at different injection rates in the middle-section model of a bifurcated channel.

Figure 18 shows that HPCF injection increased the recovery factor, sweep efficiency, and oil-displacement efficiency at both injection rates, demonstrating simultaneous improvements in macroscopic sweep and local oil mobilization. At 3 mL·min^−1^, the recovery factor increased from 26.83% to 53.30% (+26.47 percentage points), the sweep efficiency from 76.82% to 96.19% (+19.37 percentage points), and the displacement efficiency from 34.92% to 55.41% (+20.49 percentage points). The pronounced improvements indicate that the lower rate promotes prolonged slug retention and continuous resistance buildup within preferential pathways, thereby redirecting subsequent fluids toward lower-permeability and previously unswept regions.

At 4 mL·min^−1^, HPCF increased the recovery factor from 40.32% to 63.58% (+23.26 percentage points), the sweep efficiency from 87.22% to 97.23% (+10.01 percentage points), and the displacement efficiency from 46.22% to 65.39% (+19.17 percentage points). Although the final values were higher than those obtained at 3 mL·min^−1^, the incremental gains attributable to HPCF, particularly in recovery factor and sweep efficiency, were smaller. This contrast highlights the dual role of injection rate: lower rates enhance the relative flow-regulation efficiency of HPCF, whereas higher rates improve initial waterflood mobilization and final recovery. Injection-rate optimization should therefore balance slug retention, flow-diversion capacity, and the targeted development stage rather than simply maximizing injection intensity.

### 2.3. Displacement Experiment in the High-Sinuosity Channel

#### 2.3.1. Visualized Displacement Behavior

**Figure 19:**
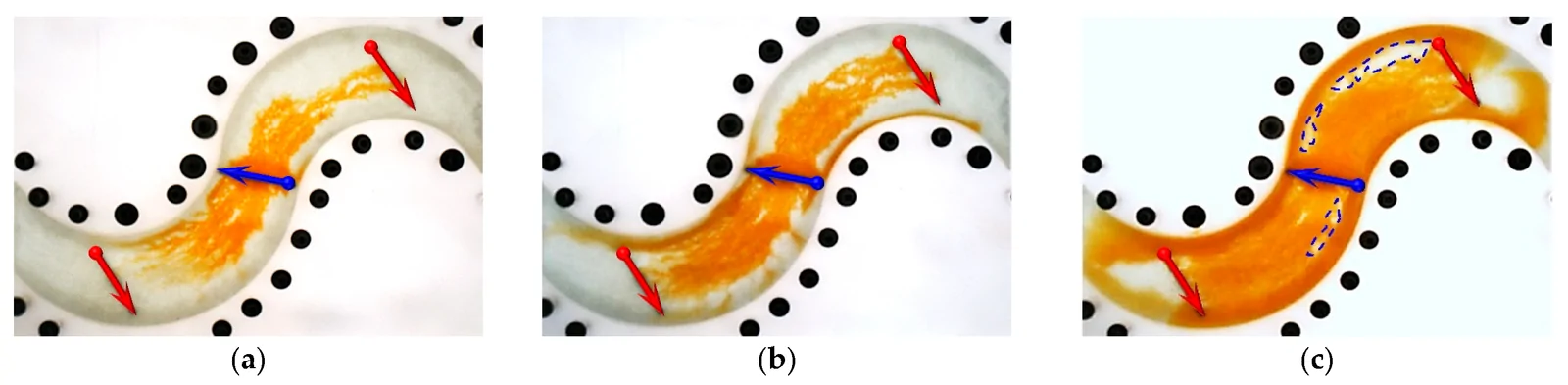
Visual results of waterflooding in the high-sinuosity channel model at 3 mL·min^−1^: (**a**) initial stage; (**b**) waterflood front breakthrough; (**c**) end of waterflooding.

**Figure 20:**
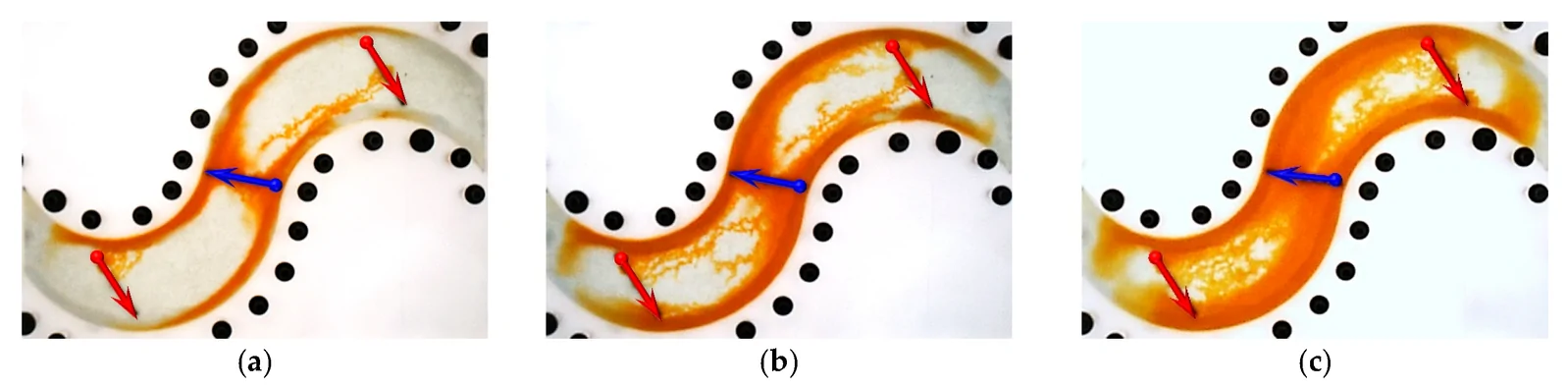
Visual results of waterflooding in the high-sinuosity channel model at 4 mL·min^−1^: (**a**) initial stage; (**b**) waterflood front breakthrough; (**c**) end of waterflooding.

In the high-sinuosity channel, displacement behavior is governed by the interaction among injection rate, permeability heterogeneity, and geometric confinement. As shown in Figure 19 and Figure 20, varying the injection rate changes not only the propagation rate and stability of the waterflood front but also the location of preferential pathways and remaining-oil accumulation.

At 3 mL·min^−1^, the front advances relatively steadily along the high-permeability channel axis. Channel curvature concentrates streamlines within the central and convex-bank regions, establishing a low-resistance connection between the injector and producer. By contrast, the concave-bank region lies outside the dominant flow direction and experiences a lower local pressure gradient and delayed front arrival, making it the principal zone of remaining-oil accumulation. At 4 mL·min^−1^, a higher pressure gradient broadens the front but also promotes localized fingering and earlier breakthrough. Geometric confinement redirects flow along the channel boundaries, generating boundary-associated preferential pathways. Although these pathways moderately enlarge the overall swept area, they reduce fluid exchange with the central region near the producer, shifting the principal remaining-oil accumulation toward this locally bypassed zone. Injection rate therefore affects both the extent and spatial distribution of sweep within the high-sinuosity channel.

**Figure 21:**
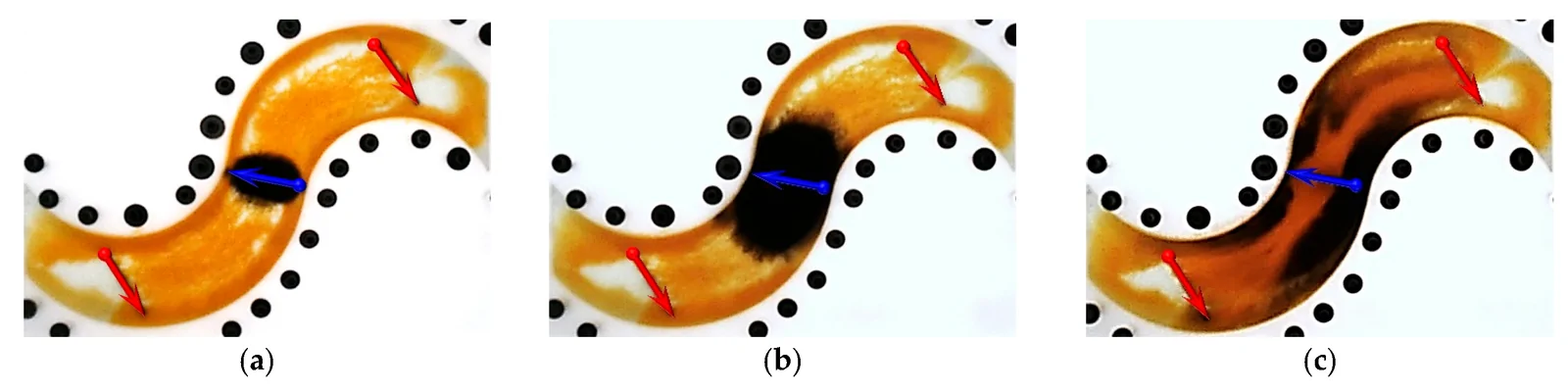
Visual results of HPCF in the high-sinuosity channel model at 3 mL·min^−1^: (**a**) initial injection stage; (**b**) end of HPCF; (**c**) end of subsequent waterflooding.

**Figure 22:**
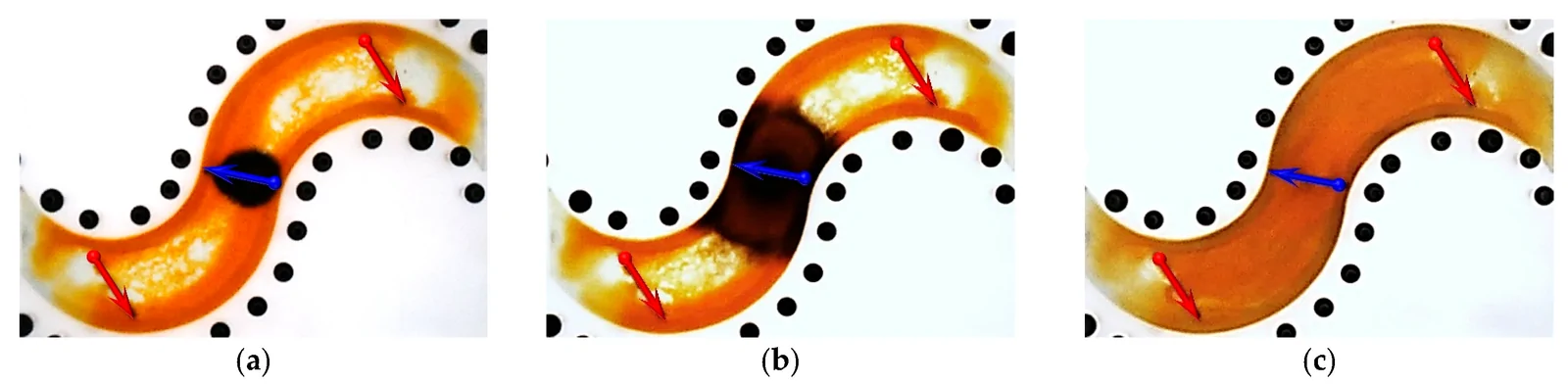
Visual results of HPCF in the high-sinuosity channel model at 4 mL·min^−1^: (**a**) initial injection stage; (**b**) end of HPCF; (**c**) end of subsequent waterflooding.

During HPCF injection, the injection rate exerts a stronger influence on slug placement and lateral coverage (Figure 21 and Figure 22). Unlike the midstream bifurcated-channel model, the high-sinuosity channel imposes pronounced curvature-induced deflection and lateral confinement. Slug transport is therefore controlled by both permeability-guided preferential flow and the ability of the imposed pressure gradient to overcome geometric resistance. At the lower injection rate, the HPCF slug remains largely confined to the pre-existing high-permeability pathways, producing an arcuate front localized along the dominant streamlines. The limited pressure gradient restricts lateral penetration into medium- and low-permeability regions, particularly those shielded by channel bends. Although B-PPG gel particles undergo retention and local accumulation within the original preferential pathways, thereby increasing flow resistance, the restricted slug coverage leaves adjacent regions insufficiently treated. Subsequent waterflooding can therefore re-establish localized breakthrough, limiting both sweep expansion and remaining-oil mobilization. At the higher injection rate, the increased pressure gradient enables the HPCF slug and B-PPG gel particles to overcome curvature-induced resistance and penetrate beyond the established preferential pathways. The slug consequently develops a broader and more uniform distribution, increasing contact with previously bypassed medium- and low-permeability regions. During subsequent waterflooding, resistance within the original pathways redirects flow toward these newly contacted zones, producing a more uniform front and greater areal sweep. The rate response of the high-sinuosity channel thus differs from that of the midstream bifurcated channel: low-rate injection favors localized retention and resistance buildup, whereas high-rate injection is required to overcome geometric confinement, broaden slug coverage, and improve overall oil mobilization. This contrast demonstrates that the optimal HPCF injection rate is configuration-dependent.

#### 2.3.2. Comparison of Displacement Performance

**Figure 23:**
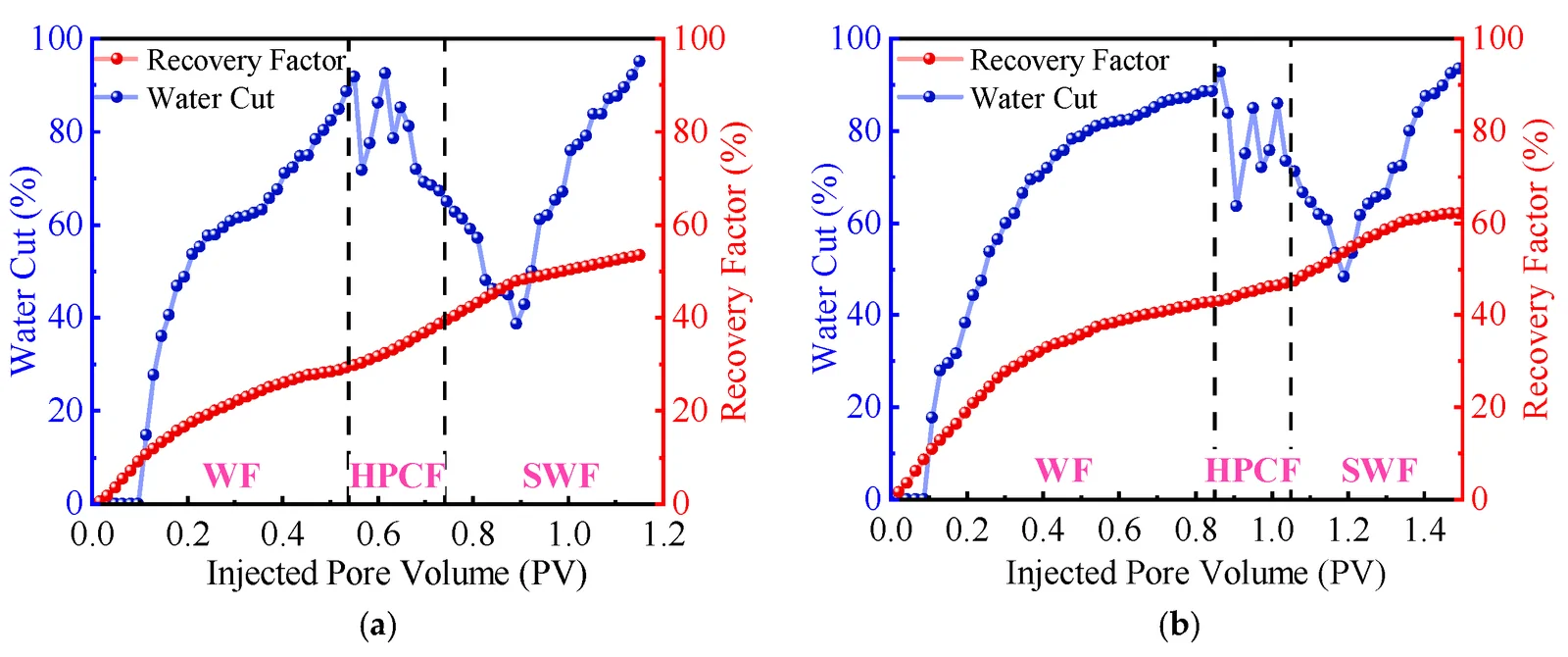
Water-cut and recovery-factor curves in the high-sinuosity channel model at different injection rates: (**a**) 3 mL·min^−1^; (**b**) 4 mL·min^−1^.

As shown in Figure 23, the water-cut and recovery-factor curves exhibit pronounced stage-dependent responses to injection rate. During initial waterflooding, the water cut follows an overall convex trend with several changes in slope. After a brief water-free production period, it rises rapidly as hydraulic communication develops between the injector and producer. The subsequent decrease in slope indicates continued lateral expansion of the displacement front before a stable preferential pathway is established. At medium to high water cut, the curve steepens again, marking the development and stabilization of preferential flow. At 3 mL·min^−1^, the comparatively slow increase in water cut indicates relatively stable frontal advancement, although the overall displacement intensity remains limited. By contrast, at 4 mL·min^−1^, injection–production communication develops more rapidly, and oil displacement is enhanced; however, the accelerated response is accompanied by reduced front stability, localized breakthrough, and earlier formation of preferential pathways.

**Figure 24:**
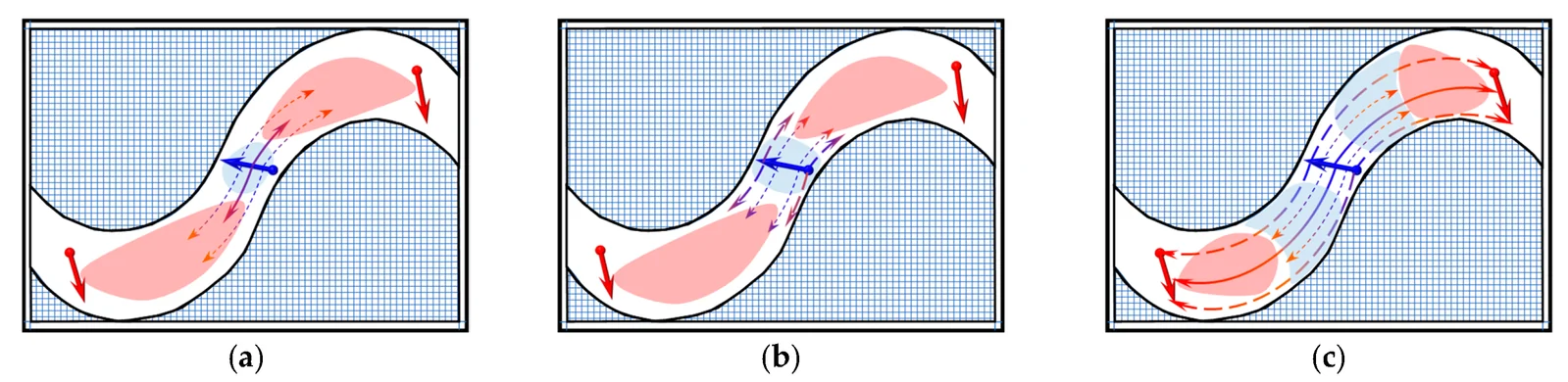
Schematic diagram of flow-field evolution during HPCF in the high-sinuosity channel model: (**a**) initial stage; (**b**) intermediate stage; (**c**) final stage.

After the transition to HPCF, both rate schemes display multipeak water-cut responses, comprising an initial decline, a transient rebound, a low-amplitude adjustment period, and a second pronounced decline (Figure 24). These successive responses reflect the coupled effects of slug placement, channel confinement, and lateral permeability heterogeneity. Initially, the HPCF slug preferentially enters the established high-permeability pathways, where B-PPG gel-particle retention and polymer-induced viscosity enhancement increase flow resistance and suppress channeling. The resulting diversion of subsequent fluid toward poorly swept regions expands the contacted volume, mobilizes bypassed oil, and lowers the produced-water fraction.

As the slug propagates into adjacent medium- to high-permeability regions, the channel-margin zones are progressively contacted, while water-rich fronts in locally high-water-cut regions continue to advance. The associated redistribution of the produced-fluid composition produces a transient rebound in water cut. Once the slug-front morphology becomes relatively stable, advancement of the marginal water front weakens, whereas hydraulic connectivity and fluid partitioning among individual flow units continue to adjust, resulting in small-amplitude water-cut fluctuations. During subsequent waterflooding, the elevated aqueous-phase viscosity and the formation of an oil-rich bank within the channel facilitate the displacement of remaining oil toward the production well. This process induces the second pronounced decrease in water cut, demonstrating that the original high-water-cut preferential-flow pattern has been effectively mitigated.

**Figure 25:**
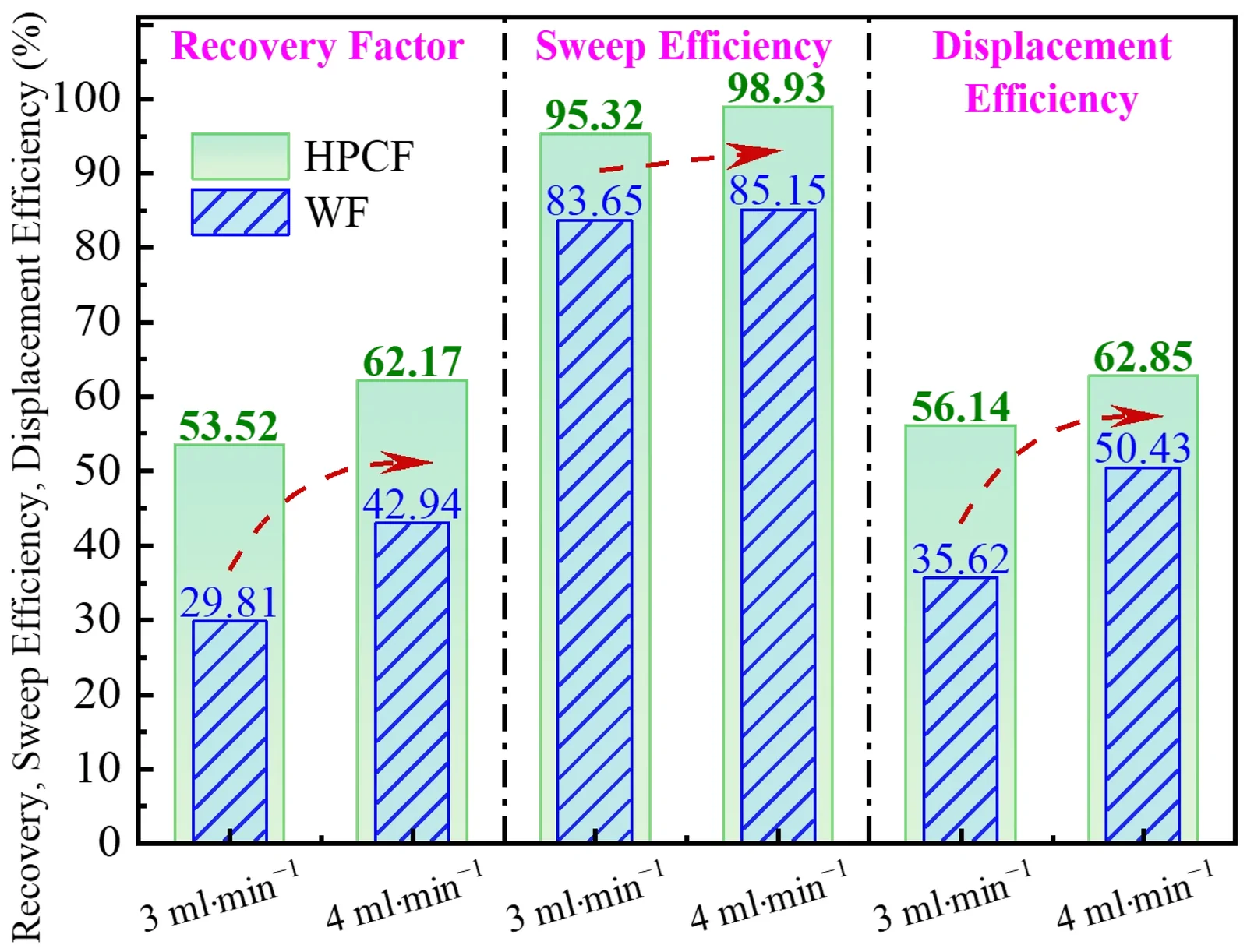
Comparison of recovery factor, sweep coefficient, and oil displacement efficiency between waterflooding and HPCF at different injection rates in the high-sinuosity channel model.

As shown in Figure 25, the recovery factor is governed by the combined contributions of the sweep coefficient and oil displacement efficiency. The performance of HPCF should therefore be evaluated at both macroscopic and microscopic scales. During waterflooding, increasing the injection rate from 3 to 4 mL·min^−1^ raises the oil displacement efficiency from 35.62% to 50.43%, whereas the sweep coefficient increases only slightly, from 83.62% to 85.15%. The higher injection rate provides a larger displacement pressure gradient and more effectively mobilizes oil at the pore scale. By contrast, the curved boundaries of the interdistributary-bay regions induce streamline deflection and lateral flow expansion, maintaining relatively high sweep coefficients at both injection rates and thereby limiting their rate-dependent difference.

HPCF substantially outperforms conventional waterflooding at both injection rates by simultaneously improving the sweep coefficient and oil displacement efficiency. At 3 mL·min^−1^, HPCF increases the recovery factor from 29.81% to 53.52%, corresponding to an increment of 23.71 percentage points. Meanwhile, the sweep coefficient increases from 83.65% to 95.32% and the oil displacement efficiency from 35.62% to 56.14%, representing gains of 11.67 and 20.52 percentage points, respectively. The larger improvement in displacement efficiency indicates that, under low-rate conditions, the incremental recovery is primarily attributable to enhanced mobilization of remaining oil, although appreciable expansion of the swept volume also occurs. Thus, the principal advantage of low-rate HPCF lies in its ability to improve the displacement of residual oil during the high-water-cut stage.

At 4 mL·min^−1^, HPCF increases the recovery factor from 42.94% to 62.17%, an increment of 19.23 percentage points. The sweep efficiency increases from 85.15% to 98.93%, whereas the displacement efficiency rises from 50.43% to 62.85%, corresponding to gains of 13.78 and 12.42 percentage points, respectively. Compared with the low-rate case, the contribution shifts toward sweep expansion. The higher pressure gradient promotes lateral slug propagation beyond the established preferential pathways, enlarging the contacted volume and producing a more spatially extensive displacement response.

The highest absolute recovery factor, sweep coefficient, and oil displacement efficiency are therefore achieved by HPCF at 4 mL·min^−1^, reaching 62.17%, 98.93%, and 62.85%, respectively. Nevertheless, the incremental recovery relative to waterflooding is greater at 3 mL·min^−1^, reflecting larger remaining-oil potential after low-rate waterflooding and more pronounced improvement in displacement efficiency induced by HPCF. Injection rate thus exerts a dual effect: low-rate injection favors efficient slug utilization and maximizes incremental recovery, whereas high-rate injection promotes sweep expansion and yields the highest ultimate recovery. Accordingly, rate optimization should account for channel geometry, the spatial distribution of remaining oil, and the targeted stage of reservoir development.

## 3. Conclusions

Based on visual heterogeneous models representing three typical narrow-channel architectures, this study elucidated the mechanisms governing flow-field evolution, preferential-channel regulation, and remaining-oil remobilization during waterflooding and HPCF treatment. The main conclusions are as follows:

1. Planform channel architecture and lateral permeability heterogeneity jointly govern flow-field evolution and the spatial distribution of remaining oil in complex narrow-channel reservoirs. Geometric confinement and permeability contrasts promote preferential-flow-pathway development and nonuniform sweep. Increasing the injection rate improves displacement locally but does not fundamentally alter the architecture-controlled flow pattern.
2. HPCF increases flow resistance within established preferential pathways, suppresses the dominance of highly conductive channels, and redirects subsequently injected fluids toward poorly swept regions. The resulting recovery enhancement arises not from simple plugging but from flow-field reconfiguration through the synergistic effects of particle retention, polymer-induced viscosity enhancement, and selective resistance increase. This process expands the effective swept area and promotes remaining-oil remobilization.
3. The flow-profile-control efficacy of HPCF strongly depends on channel architecture and operating conditions. Planform architecture determines the locations of preferential pathways, the principal zones of HPCF intervention, and the trajectories of diverted flow. Well-pattern configuration and injection rate further regulate HPCF transport and retention and, consequently, the spatial extent and magnitude of flow-field reconfiguration.
4. Across the three representative channel architectures, HPCF injection increased the recovery factor by an additional 19.23–26.47 percentage points after waterflooding, demonstrating its effectiveness under high-water-cut conditions. Late-stage development strategies should therefore move beyond simply increasing injection intensity and instead adopt architecture-specific optimization of HPCF-slug design, injection parameters, and well-pattern configuration according to preferential-flow-pathway development and remaining-oil distribution.

## 4. Materials and Methods

### 4.1. Geological Abstraction and Scaling Laws

#### 4.1.1. Characteristics of Typical Narrow-Channel Configurations

**Figure 26:**
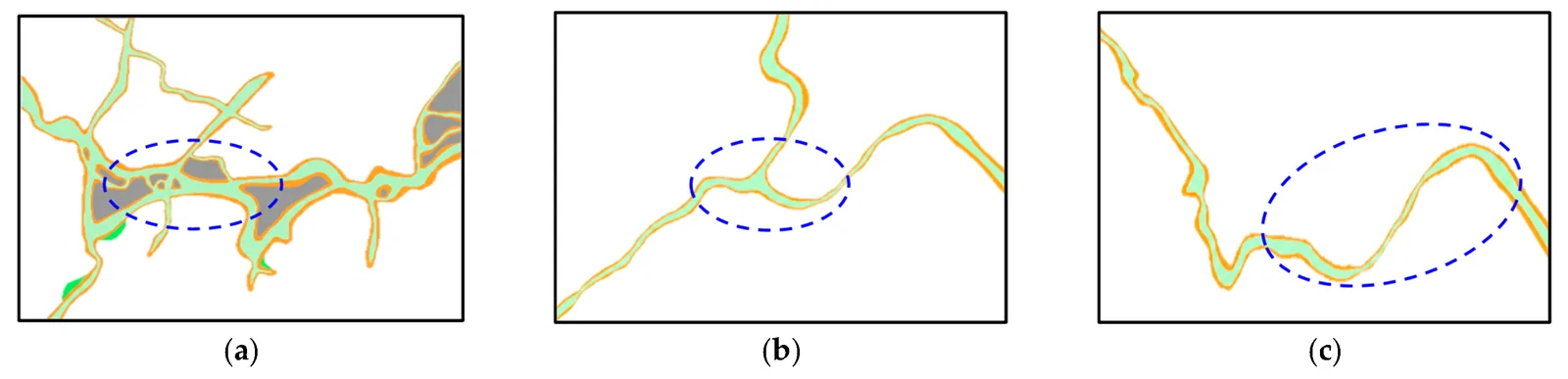
Planform characteristics of typical narrow channel configurations. (**a**) Front section of a bifurcated channel; (**b**) middle section of a bifurcated channel; (**c**) high-sinuosity channel.

Narrow-channel reservoirs are shaped by channel migration, bifurcation and reconstruction, and multistage channel stacking, resulting in narrow-belt geometries, variable interbranch connectivity, and pronounced lateral heterogeneity. To establish physical models representing the dominant reservoir architectures in the Bozhong block, complex channelized sand bodies were conceptually simplified on the basis of their planform distributions, sedimentary-facies interpretations, and detailed reservoir-architecture analyses. Channel geometry, branch connectivity, cross-cutting and superposition relationships, and sinuosity were selected as the principal classification criteria. Three representative configuration types were identified: the front section of a bifurcated channel, the middle section of a bifurcated channel, and a high-sinuosity channel (Figure 26).

The front section of a bifurcated channel exhibits a characteristic Y-shaped geometry, restricted interbranch connectivity and significant lateral facies transitions. These features promote the development of relatively isolated flow compartments, resulting in asynchronous production responses and water breakthrough among individual branches. The middle section of a bifurcated channel is characterized by X-shaped channel intersections and superposition. Multistage channel stacking enhances local sand-body connectivity and favors flow concentration and preferential flow pathways. The high-sinuosity channel exhibits an S-shaped narrow-belt geometry, with strong constraints imposed by channel boundaries. Fluid migration is significantly affected by curvature variations and lateral boundary controls, which may induce streamline deviation, local water fingering, and residual oil trapping near the channel margins. The above three architectural patterns represent three typical geological control mechanisms: branch connectivity, cross-cutting superposition, and curved boundary constraints. They provide geological prototypes for subsequent 2D physical model design and the analysis of flow-field evolution controlled by reservoir architectures.

#### 4.1.2. Basis of Experimental Design

(1) Experimental Model Design

Following the identification of representative architectural patterns, the natural narrow-channel bodies were abstracted into 2D visual physical models. Model construction followed the principles of preserving dominant architectures, simplifying local geological details, and highlighting architecture-dependent flow behavior. Key geometric attributes, including channel length and width, branching morphology, intersection patterns, sinuosity, and major lateral boundaries, were retained, while secondary sand bodies, local pinch-outs, small-scale facies variations, and minor boundary irregularities were simplified. This abstraction was not intended to reproduce the full geological complexity of the reservoir but to isolate the dominant effects of channel architectures on waterflood front propagation, preferential flow pathway development, and residual oil distribution under controlled conditions.

**Figure 27:**
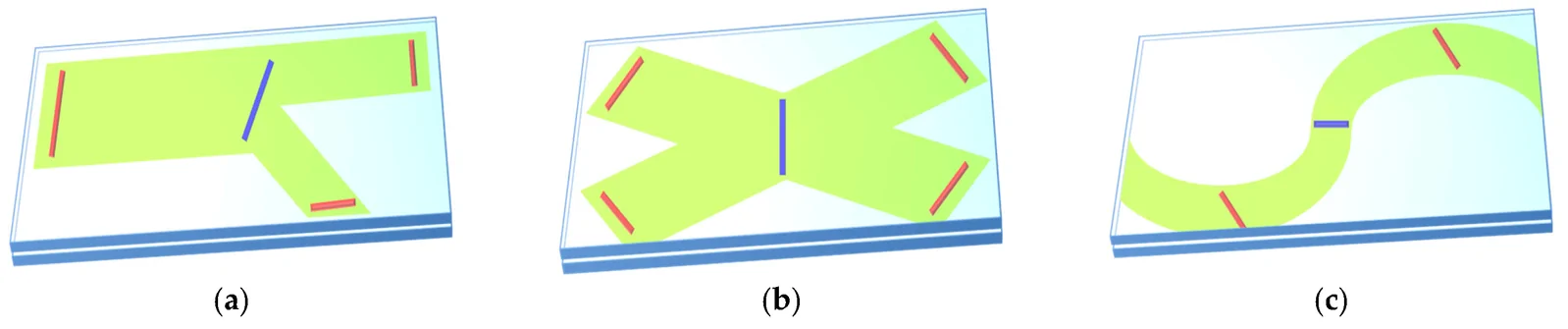
Design of 2D sand-packed models with different channel configurations. (**a**) Front-section model of a bifurcated channel; (**b**) middle-section model of a bifurcated channel; (**c**) high-sinuosity channel model.

Three representative models were constructed. The front-section model of a bifurcated channel preserved a Y-shaped branching geometry with restricted interbranch connectivity and was designed to characterize distal branch segments, marginally swept regions, and asynchronous water breakthrough. The middle-section model of a bifurcated channel retained X-shaped channel intersections and superposition relationships and was mainly used to investigate preferential flow pathway development in multi-branch intersection zones, uneven flow distribution, and local residual oil enrichment. The highly sinuous model preserved an S-shaped main channel and narrow-belt boundary constraints, enabling examination of streamline deviation, localized water channeling, and curvature-induced residual oil trapping near channel margins. Through the above design, the three models captured distinct channel geometries, connectivity patterns, and boundary constraints, providing a unified experimental basis for comparing flow-field responses during waterflooding and HPCF injection under different architectural conditions (Figure 27).

(2) Design of Similarity Criteria

To reproduce the dominant multiphase flow behavior of the target reservoir (80 °C and 12.56 MPa) under ambient laboratory conditions (26 °C and 0.1 MPa), similarity criteria for the 2D physical simulation were established using dimensional analysis and engineering similarity theory. Given the substantial differences in temperature, pressure, spatial scale, and boundary conditions between the experimental model and the reservoir, strict geometric scaling of all parameters was not pursued. Instead, an equivalent design was adopted to preserve the principal factors governing waterflood front propagation, preferential flow pathway development, and residual oil distribution. Particular emphasis was placed on maintaining similarity in channel planform geometry, permeability contrast, oil–water mobility ratio, and injection intensity. Accordingly, the model dimensions, porous-medium properties, fluid viscosity, and injection rate were adjusted to reproduce the first-order flow-field evolution and dominant displacement mechanisms of complex narrow-channel reservoirs. The surface chemistry of the glass beads differs from that of natural sandstone. Consequently, the experimental results primarily characterize the macroscopic displacement and flow-regulation behavior of the HPCF system and should not be directly extrapolated to quantify chemical adsorption or retention in natural sandstone reservoirs.

The experimental design was developed in terms of geometric, kinematic, and dynamic similarity. For geometric similarity, the model preserved the planform geometries of representative narrow channels, including branch connectivity, curved channel boundaries, and the principal spatial relationships between injection and production wells. This design enabled the effects of channel architecture on waterflood-front propagation and HPCF-slug migration to be systematically evaluated. For kinematic similarity, the effects of the temperature difference between the laboratory and reservoir conditions on aqueous-phase viscosity and the oil–water mobility ratio were considered. A model oil with a viscosity of 35 mPa·s was therefore prepared to approximate an oil–water mobility ratio, thereby reproducing the dominant characteristics of preferential flow pathway development and nonuniform fluid advancement during the high-water-cut stage. For dynamic similarity, the injection rate was set at 3.0–4.0 mL·min^−1^ on the basis of injection-intensity and capillary-number similarity, ensuring comparable displacement-front propagation and macroscopic sweep behavior.

**Figure 28:**
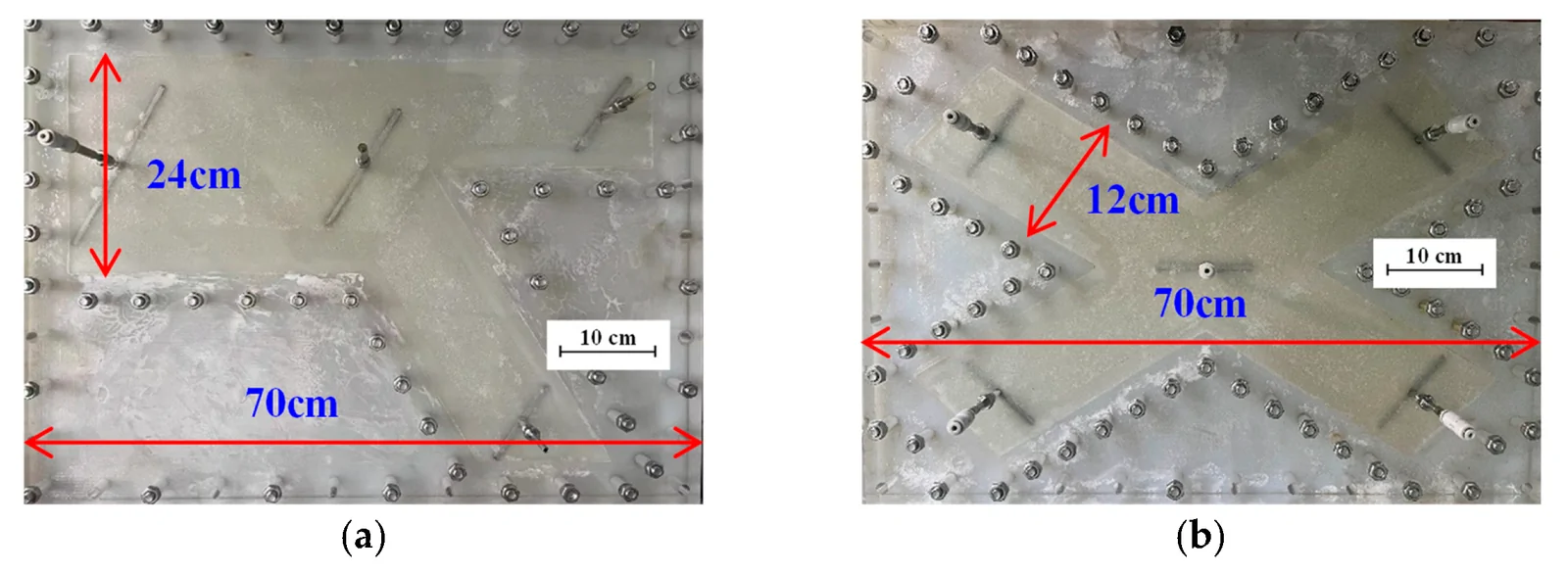
2D visualized sand-packed models. (**a**) Front-section model of a bifurcated channel; (**b**) middle-section model of a bifurcated channel.

The 2D physical model was not intended to reproduce the complete 3D architecture or in situ thermodynamic conditions of the reservoir. Instead, it served as a reduced-order physical analog that preserved the dominant channel-planform geometries, permeability contrasts, and oil–water mobility characteristics. The design was therefore used to isolate the effects of channel architecture, injection rate, and well-pattern configuration on preferential flow pathway development, flow-field reconfiguration induced by HPCF injection, and residual oil remobilization. The model dimensions, fluid properties, and injection-production parameters, together with their corresponding reservoir values, are summarized in Table 1, and the physical model is shown in Figure 28.

**Figure 29:**
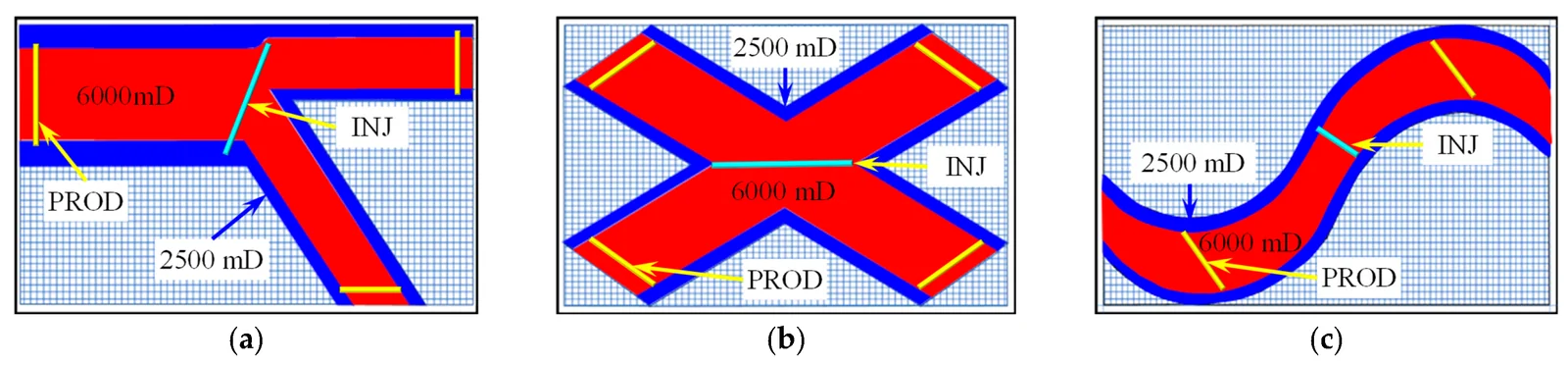
Permeability distribution of the 2D sand-packed model. (**a**) Front-section model of a bifurcated channel; (**b**) middle-section model of a bifurcated channel; (**c**) high-sinuosity channel model.

To capture the areal heterogeneity of narrow-channel sand bodies, in which permeability progressively decreases from the channel axis toward its margins, a zonal sand-packing scheme was implemented in the two-dimensional physical model (Figure 29). The continuous lateral permeability variation observed in natural channel deposits was discretized into a high-permeability central zone flanked by low-permeability marginal zones. This idealization was not intended to reproduce the full complexity of reservoir heterogeneity but to isolate the first-order effects of the lateral permeability gradient and channel-boundary constraints on waterflood-front propagation and residual oil distribution.

### 4.2. Experimental Materials

Simulated water: Ultrapure water was used as both the saturation and displacement water.

Model oil: The model oil was prepared by blending industrial lubricating oil with kerosene. At 26 °C, it had a viscosity of 35 mPa·s and a density of 0.83 g·cm^−3^.

Partially hydrolyzed polyacrylamide (HPAM) : The HPAM (Shandong Juxing Petroleum Technology Co., Ltd, Dongying, China) had a molecular weight of 2.0 × 10^7^, a solid content of 89.55%, and a degree of hydrolysis of 20.4%. The HPAM solution was prepared in ultrapure water at a concentration of 1000 mg·L^−1^.

Surfactant: The surfactant (Zhongsheng Environmental Protection Co., Ltd., Dongying, China) was a petroleum sulfonate with the general molecular formula C_23_H_38_SO_3_M.

Bulk-swelling preformed particle gel (B-PPG): The B-PPG (Shandong Juxing Petroleum Technology Co., Ltd., Dongying, China) had an elastic modulus of 10.3 Pa and a particle size of 100–150 mesh. The B-PPG was dispersed in the ultrapure water as a dry powder and swelled upon hydration to form gel particles, which preferentially entered high-permeability flow pathways and increased flow resistance, thereby improving displacement performance.

Glass beads: Borosilicate glass beads (Xinhui Abrasives Co., Ltd., Foshan, China) with a particle size range of 60–100 mesh and a mean diameter of approximately 150 μm were used to construct the porous medium. Before each experiment, the beads were thoroughly rinsed with ultrapure water to remove surface impurities and then dried at 120 °C to ensure reproducible surface and wettability conditions. Because untreated borosilicate glass is intrinsically hydrophilic, the resulting porous medium was water-wet.

Dyes: Methyl orange (Huasheng Chemical Reagent Co., Ltd., Tianjin, China) and methyl blue (Huasheng Chemical Reagent Co., Ltd., Tianjin, China) were used to distinguish the displacement water and the HPCF system, respectively.

To construct an HPCF system integrating mobility control, interfacial regulation, and preferential-flow-path regulation, HPAM, petroleum sulfonate surfactant, and B-PPG were dissolved in simulated formation water at prescribed concentrations. In this system, HPAM primarily improves the oil–water mobility ratio by increasing the aqueous-phase viscosity, while the petroleum sulfonate surfactant enhances oil displacement by reducing the oil–water interfacial tension. B-PPG, as the key particulate gel component of the system, is mainly responsible for regulating preferential flow pathways and inducing fluid diversion. Unlike conventional in-situ crosslinked weak gels, B-PPG has a stable crosslinked network structure prior to injection, and the gel particles exhibit favorable water-swelling capacity, elastic deformability, and transport stability. After entering the porous medium, B-PPG particles preferentially migrate into high-permeability, low-resistance flow pathways and progressively increase the flow resistance of dominant channels through particle migration, elastic deformation, retention, and local accumulation. Under sustained injection, the particles can undergo compressive deformation and pass through certain pore throats, followed by re-expansion and redistribution in downstream regions, thereby forming a dynamic “migration–retention–remigration” process with a certain degree of in-depth flow-regulation capability. This process does not simply result in permanent plugging; instead, it selectively increases flow resistance in preferential pathways, weakens the established water-channeling paths, and redirects subsequent displacement fluids toward insufficiently swept regions, thereby redistributing the flow field. Therefore, in the HPCF system, B-PPG functions not only as a particulate gel agent for flow regulation but also as an important functional component linking macroscopic flow-field reconstruction with remaining-oil mobilization, while acting synergistically with the mobility-control effect of HPAM and the interfacial-regulation effect of the surfactant. The base formulation adopted in this study was 1000 mg·L^−1^ HPAM + 1000 mg·L^−1^ surfactant + 500 mg·L^−1^ B-PPG, as shown in Table 2.

### 4.3. Experimental Apparatus and Data Acquisition

**Figure 30:**
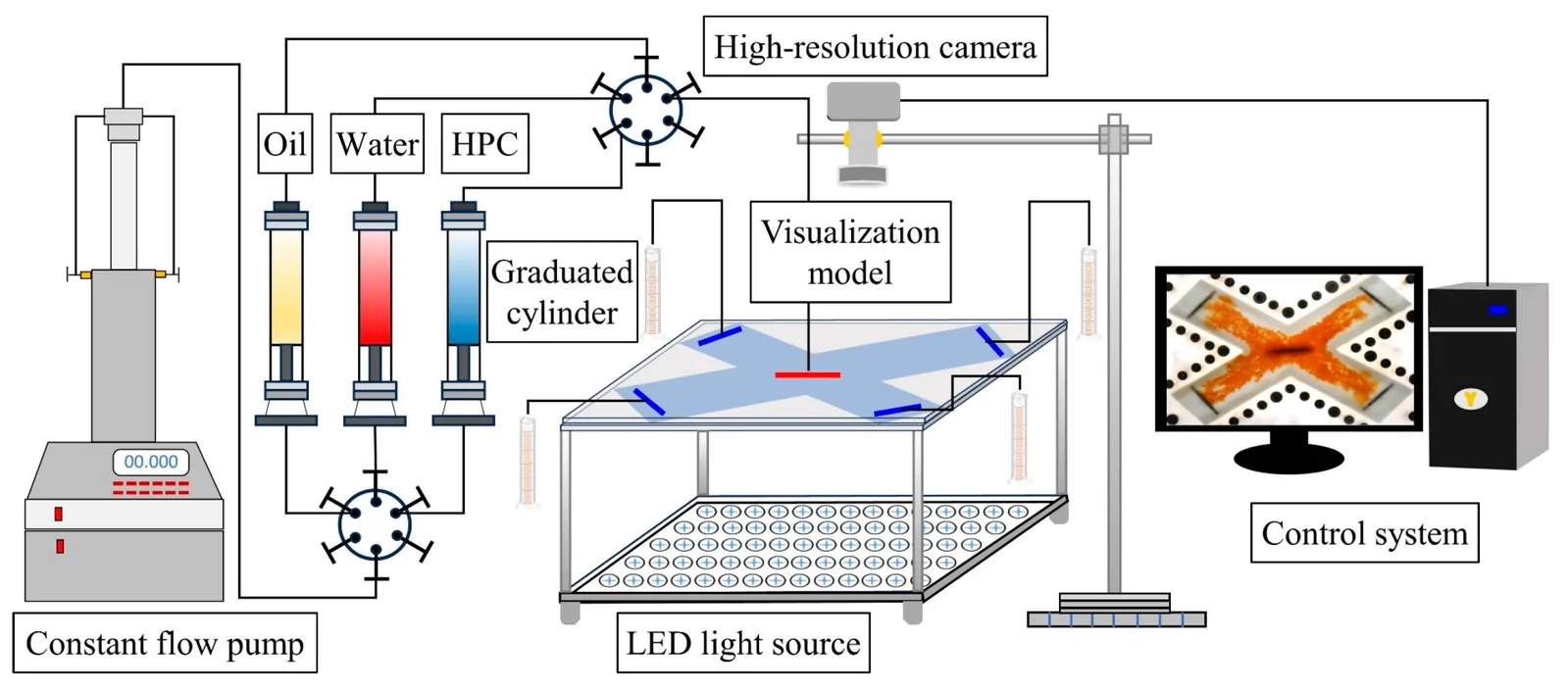
Schematic diagram of the experimental apparatus.

Figure 30 schematically illustrates the experimental apparatus, which comprised three principal modules: a fluid-injection system, a two-dimensional visual displacement system, and a data-acquisition system. The principal components included an ISCO dual-cylinder syringe pump, an intermediate fluid vessel, a visual physical model, a produced-fluid collection unit, and a real-time imaging system. The ISCO pump provided constant-rate injection and precise pressure control over a flow-rate range of 0.01–25.00 mL·min^−1^, with a maximum operating pressure of 689.5 bar. These specifications ensured stable and continuous injection during both waterflooding and chemical-slug injection.

The visual physical model constituted the core of the experimental system. It measured 35 cm × 35 cm × 3 cm, with horizontal wells arranged according to the prescribed narrow-channel geometries and well-pattern configurations. A transparent acrylic cover plate enabled real-time visualization of the advancing waterflood front, the evolution of the swept region, and the spatial distribution of residual oil. Leak-tightness was achieved using a peripheral rubber gasket coated with silicone sealant. The model was compressed between upper and lower steel plates using 56 through-bolts, ensuring reliable sealing and mechanical stability at the operating pressure of 1 MPa. During each experiment, the effluent from the model outlet was quantitatively collected at 60 s intervals. The collected oil–water mixtures were sealed and heated in a water bath until complete phase separation and stabilization of the oil–water interface was achieved. The volumes of oil and water phases were then measured separately to determine the recovery factor and water cut. Concurrently, the real-time imaging system continuously captured the evolution of fluid distribution within the model, providing image data for subsequent characterization of flow-field dynamics and quantitative analysis of residual oil distribution.

### 4.4. Experimental Design and Procedures

(1) Experimental Preparation

**Figure 31:**
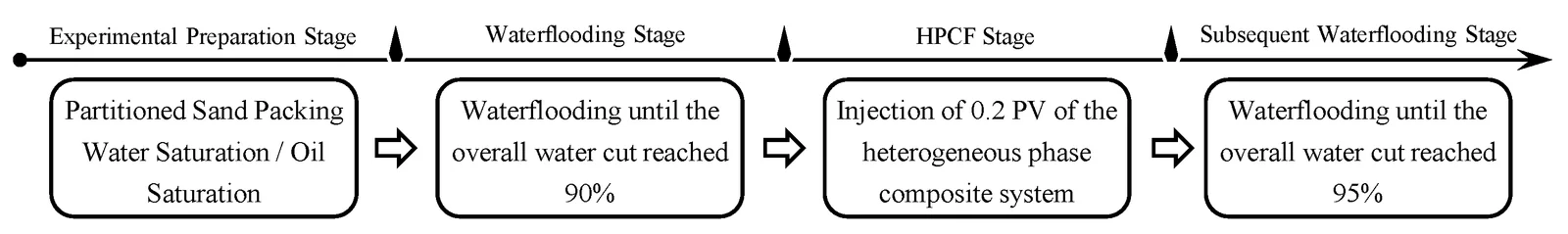
Experimental procedure.

Glass beads of different particle sizes were washed with ultrapure water and dried at 120 °C for 12 h to remove surface organic contaminants and ensure consistent initial wettability. The experimental procedure is shown in Figure 31.

Reservoir heterogeneity was represented using the zonal sand-packing approach. During model preparation, the packed glass beads were compacted by mechanical vibration to achieve a stable pore structure. The transparent acrylic top plate was then installed, and the bolts were tightened to ensure effective sealing.

The experimental apparatus was assembled and tested for airtightness. After confirming that the system was well sealed, the model was evacuated using a vacuum pump.

Ultrapure water was injected at a constant rate of 3 mL·min^−1^ until full water saturation was achieved. The pore volume (PV) and porosity of the model were determined using the material balance method, after which the model was allowed to equilibrate for 4 h to stabilize the fluid distribution.

Simulated oil was injected at a constant rate of 2 mL·min^−1^ until no further water was produced at the outlet. The cumulative injected oil volume was recorded to determine the original oil in place (OOIP), after which the model was allowed to equilibrate for 24 h to stabilize the fluid distribution.

(2) Experimental Procedure

Baseline waterflooding stage: The model was maintained at room temperature, with the experimental temperature controlled at 26 °C. During waterflooding, water was injected into the injection well at a constant rate of 3 mL·min^−1^ until the overall water cut reached 90%, after which the experiment proceeded to the subsequent stage.

Slug injection stage: After completion of the baseline waterflooding, a 0.2 PV HPCF slug was injected. The slug consisted of 1000 mg·L^−1^ HPAM, 1000 mg·L^−1^ surfactant, and 500 mg·L^−1^ B-PPG. Subsequent waterflooding was then conducted at the same injection rate.

Subsequent waterflooding stage: Waterflooding was continued at the same injection rate (3 mL·min^−1^). The experiment was terminated when the overall water cut at the production well reached 97%.

To clarify the multiphase flow behavior and residual oil mobilization mechanisms in complex narrow-channel reservoirs, heterogeneous visualized physical models were constructed based on the three typical channel architectures described above, namely the front section of a bifurcated channel, the middle section of a bifurcated channel, and the high-sinuosity channel. With the macroscopic geological architectures as the basis, the injection-production well pattern and injection rate were introduced as the principal engineering variables, and a multifactor displacement scheme based on the control-variable method was designed, as shown in Table 3. The entire physical simulation was conducted strictly according to the sequence of baseline waterflooding, HPCF slug injection, and subsequent waterflooding to elucidate flow-field evolution and the mechanisms responsible for sweep-efficiency enhancement.

### 4.5. Quantitative Analysis Based on Image Processing

To address the irregular advancement of fluid fronts, indistinct phase-interface transitions, and substantial variations in residual-oil patch size observed during visualized displacement experiments in complex narrow-channel reservoirs, a quantitative flow-field analysis method based on image processing and semantic segmentation was developed. The method comprised four steps: image preprocessing, color-feature segmentation, post-processing correction, and quantitative parameter extraction. First, the original experimental images were subjected to image registration, background subtraction, and illumination correction to reduce the effects of model boundaries, sand-pack textures, and local illumination nonuniformity on the recognition results. Subsequently, RGB-to-CIELAB color-space transformation was combined with pixel-level classification to identify the swept regions of the displacing fluid, residual-oil regions, and nonfluid background regions. To improve the stability of identifying indistinct displacement fronts and fragmented oil patches, the segmentation results were further corrected through boundary enhancement, connected-component filtering, and morphological processing. Finally, the swept area and residual-oil area were calculated from the corrected segmentation masks, and quantitative indicators, including the sweep coefficient and displacement efficiency, were subsequently determined. These indicators provided a basis for evaluating the expansion of the swept region and the remobilization of residual oil induced by HPCF.

**Figure 32:**
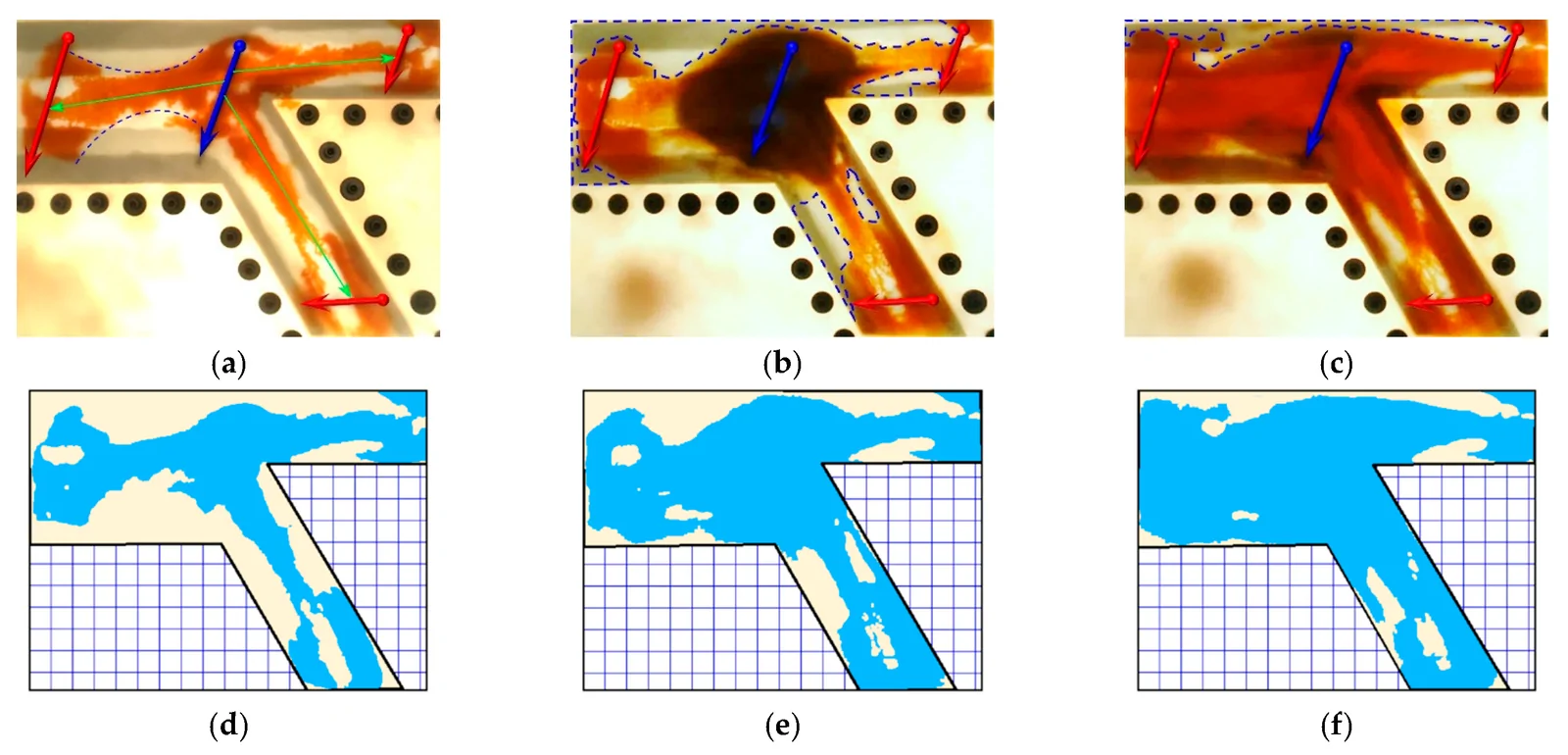
Validation of experimental image processing results. (**a**) End of waterflooding; (**b**) end of HPCF; (**c**) end of subsequent waterflooding; (**d**) processed image after waterflooding; (**e**) processed image after HPCF; (**f**) processed image after subsequent waterflooding.

As shown in Figure 32, taking the image-processing results at representative displacement stages as examples, the proposed workflow clearly distinguished the swept regions of the displacing fluid, unswept regions, and residual-oil distribution. The processed binary images largely preserved the morphology of the displacement front and the principal characteristics of the residual-oil patches, thereby providing an image basis for the subsequent calculation of swept area, residual-oil area, and related quantitative parameters.

Based on the validated image-analysis results described above, a quantitative model for calculating the sweep coefficient and displacement efficiency was established by mapping image pixel information to the physical domain.

The sweep coefficient in the model was defined as the ratio of the area swept by the displacing fluid to the total model area and was calculated as follows:

(1) $$E_{A}=\frac{A_{swept}}{A_{total}}=\frac{\sum_{i=1}^{n}P_{s,i}\times \Delta S}{\sum_{j=1}^{N}P_{t,j}\times \Delta S}\times 100\%$$

where *A_swept_* is the area swept by the displacing fluid, either water or the HPCF system, cm^2^; *A_total_* is the total effective pore area of the model, cm^2^; *P_s,i_* is the total number of pixels identified as the swept region after image segmentation; *P_t,j_* is the total number of effective pixels within the model; and Δ*S* is the actual physical area represented by a single pixel, cm^2^·pixel^−1^.

Furthermore, by integrating the material-balance data recorded in real time at the production outlet, the actual oil-displacement capability within a specific swept region could be back-calculated. Based on the relationship among oil recovery *R_i_*, the sweep coefficient, and displacement efficiency, the displacement efficiency was calculated as follows:

(2) $$E_{D,i}=\frac{R_{i}}{E_{A,i}}=\frac{V_{o,i}/V_{oi}}{E_{A,i}}\times 100\%$$

where *E_D_*_,_*_i_* is the average displacement efficiency during the *i* displacement stage, %; *R_i_* is the cumulative oil recovery at that stage, %; *V_o,i_* is the cumulative volume of oil produced by that stage, cm^3^; and *V_oi_* is the original oil in place initially saturated in the physical model, cm^3^.

## Figures and Tables

**Table 1:** Comparison of original reservoir parameters and model parameters.

Parameter	Actual Reservoir Data	Simulation Model Data
Reservoir Temperature	80 °C	26 °C
Reservoir Pressure	12.56 MPa	0.1 MPa
Porosity	0.31	0.30
Permeability	3650 mD	3500 mD
Formation Oil Density	0.89 g∙cm^−3^	0.86 g∙cm^−3^
Formation Oil Viscosity	35 mPa∙s	35 mPa∙s
Formation Water Density	1.02 g∙cm^−3^	1.02 g∙cm^−3^
Formation Water Viscosity	0.46 mPa∙s	0.96 mPa∙s
HPCF Viscosity	50.4 mPa∙s	50.4 mPa∙s
Channel Length	400 m	24 cm
Channel Width	200 m	12 cm
Wellbore Diameter	0.138 m	0.3 cm
Injection Rate	120–450 m^3^∙d^−1^	1.5–4.1 mL·min^−1^

**Table 2:** Specific formulation and component parameters of the HPCF system.

**Solution Name**	**Solution Formulation**	**Solution Concentration (mg·L** **^−1^ )**	**Solution Color**
Heterogeneous-Phase Composite System	HPAM	1000	Blue
	Surfactant	1000	
	B-PPG	500	

**Table 3:** Design of physical simulation experiments.

Experimental Group	No.	Channel Type	Well Pattern	Injection Rate mL·min^−1^	Experimental Stage
Comparison of Injection Rates	1	Front-End Branched Channel Model	1I-3P	3	WF
	2		1I-3P	4	
Comparison of Well Pattern Optimization	3		1I-3P	3	WF + HPCF
	4		3I-1P		
Comparison of Injection Rates	5	Mid-Reach Branched Channel Model	1I-4P	3	WF + HPCF
	6			4	
Comparison of Injection Rates	7	High-sinuosity Channel Model	1I-2P	3	WF + HPCF
	8			4	

## Data Availability

The data supporting the findings of this study are available from the corresponding author upon reasonable request.

## Abbreviations

The following abbreviations are used in this manuscript:

WF	Waterflooding
HPCF	Heterogeneous-phase composite flooding
SWF	Subsequent Waterflooding

## References

[1] Sun Z., Bai Q., Ren H., Yan K., Gao L., Liu Z.W. (2021). Quantitative evaluation of morphological characteristics of thin sand body: Taking the deltaic distributary channel sand body as an example in Shengtuo oil field of Dongying Sag, east of Bohai Bay Basin. J. Pet. Sci. Eng..

[2] Xu Z., Wu S., Liu Z., Zhao J., Geng H., Wu J., Zhang T., Liu Z. (2019). Sandbody architecture of the bar finger within shoal water delta front: Insights from the Lower Member of Minghuazhen Formation, Neogene, Bohai BZ25 Oilfield, Bohai Bay Basin, East China. Pet. Explor. Dev..

[3] Ma K., Wang J., Si S., Yang L., Zhao Z., Ling H., Wu Z. (2025). Study on three-dimensional physical simulation experiment of remaining oil distribution in narrow channel reservoir. Front. Earth Sci..

[4] Jia P., Li Y., Liu B., Feng H. (2025). Practical streamline method for quantitatively characterizing waterflooding in narrow channel reservoirs. ACS Omega.

[5] Yu C., Zhang M., Chen W., Zhang S., Wang S. (2024). A microscopic experimental study on the dominant flow channels of water flooding in ultra-high water cut reservoirs. Energies.

[6] Jia P., Sun Q., Feng H., Li Y., Liu B. (2025). Experimental study on microscopic residual oil distribution and dredging direction of water-driven zoned utilization in narrow-channel reservoirs. Front. Earth Sci..

[7] Zheng X., Wang Y., Lei Y., Zhang D., Bao W., Huang S. (2025). Research and application of deep profile control technology in narrow fluvial sand bodies. Processes.

[8] Seright R.S., Brattekas B. (2021). Water shutoff and conformance improvement: An introduction. Pet. Sci..

[9] Zhang X., Yu J., Liu L., Liu X., Lu X., Feng Q. (2025). Experimental investigation of synergistic enhanced oil recovery by infill well pattern and chemical flooding after polymer flooding. Gels.

[10] Du Q.J., Pan G.M., Hou J., Guo L.L., Wang R.-R., Xia Z.Z., Zhou K. (2019). Study of the mechanisms of streamline-adjustment-assisted heterogeneous combination flooding for enhanced oil recovery for post-polymer-flooded reservoirs. Pet. Sci..

[11] Wu D., Zhou K., Hou J., An Z., Zhai M., Liu W. (2020). Experimental study on combining heterogeneous phase composite flooding and streamline adjustment to improve oil recovery in heterogeneous reservoirs. J. Pet. Sci. Eng..

[12] Zhang J., Li Y., Li X., Guan C., Chen X., Liang D. (2024). Mechanism of enhanced oil recovery by discontinuous chemical flooding in Bohai oilfield. Acta Pet. Sin..

[13] Li P., Shi L., Wang X., Gao H., Ge S., Wang S. (2025). Research on the “multi branch relay” chemical flooding mode for improving oil recovery in high water content and high heterogeneity reservoirs. Geoenergy Sci. Eng..

[14] Mohsenatabar Firozjaii A., Saghafi H.R. (2020). Review on chemical enhanced oil recovery using polymer flooding: Fundamentals, experimental and numerical simulation. Petroleum.

[15] Zhu Y., Hou Q., Jian G., Ma D., Wang Z. (2013). Current development and application of chemical combination flooding technique. Pet. Explor. Dev..

[16] Bai B., Liu Y., Coste J.-P., Li L. (2007). Preformed particle gel for conformance control: Transport mechanism through porous media. SPE Reserv. Eval. Eng..

[17] Leng J., Wei M., Bai B. (2021). Review of transport mechanisms and numerical simulation studies of preformed particle gel for conformance control. J. Pet. Sci. Eng..

[18] Mehrabianfar P., Malmir P., Soulgani B.S., Hashemi A. (2020). Study on the optimization of the performance of preformed particle gel (PPG) on the isolation of high permeable zone. J. Pet. Sci. Eng..

[19] Saghafi H.R., Emadi M.A., Farasat A., Arabloo M., Naderifar A. (2016). Performance evaluation of optimized preformed particle gel (PPG) in porous media. Chem. Eng. Res. Des..

[20] Cao X., Liu Y., Cao W. (2022). Matching relation between preformed particle gel and reservoir pore throat during heterogeneous phase combination flooding. Acta Pet. Sin..

[21] Li J., Jiang Z., Wang Y., Zheng J., Huang G. (2018). Stability, seepage and displacement characteristics of heterogeneous branched-preformed particle gels for enhanced oil recovery. RSC Adv..

[22] Imqam A., Bai B. (2015). Optimizing the strength and size of preformed particle gels for better conformance control treatment. Fuel.

[23] Tongwa P., Bai B. (2014). Degradable nanocomposite preformed particle gel for chemical enhanced oil recovery applications. J. Pet. Sci. Eng..

[24] Ye R., Wang L., Xu W., Zhang J., Chen Z. (2024). Micro and macro flooding mechanism and law of a gel particle system in strong heterogeneous reservoirs. Gels.

[25] Ma S., Dong M., Li Z., Shirif E. (2007). Evaluation of the effectiveness of chemical flooding using heterogeneous sandpack flood test. J. Pet. Sci. Eng..

[26] Zhou X., Dong M., Maini B. (2013). The dominant mechanism of enhanced heavy oil recovery by chemical flooding in a two-dimensional physical model. Fuel.

[27] He H., Fu J., Zhao H., Yuan F., Guo L., Li Z., Wang X., Peng H. (2018). Synergistic mechanism of hydrolyzed polyacrylamide enhanced branched-preformed particle gel for enhanced oil recovery in mature oilfields. Energy Fuels.

[28] Gong H., Zhang H., Xu L., Li K., Yu L., San Q., Li Y., Dong M. (2017). The synergistic effect of branched-preformed particle gel and hydrolyzed polyacrylamide on further-enhanced oil recovery after polymer flooding. Energy Fuels.

[29] Gao Q., Zhong C., Han P., Cao R., Jiang G. (2020). Synergistic effect of alkali–surfactant–polymer and preformed particle gel on profile control after polymer flooding in heterogeneous reservoirs. Energy Fuels.

[30] He H., Fu J., Hou B., Yuan F., Guo L., Li Z., You Q. (2018). Investigation of injection strategy of branched-preformed particle gel/polymer/surfactant for enhanced oil recovery after polymer flooding in heterogeneous reservoirs. Energies.

[31] Zhang X., Zhang Y., Liu H., Li S., Liu L. (2023). Dynamic sweep experiments on a heterogeneous phase composite system based on branched-preformed particle gel in high water-cut reservoirs after polymer flooding. Gels.

[32] Alhuraishawy A.K., Sun X., Bai B., Wei M., Imqam A. (2018). Areal sweep efficiency improvement by integrating preformed particle gel and low salinity water flooding in fractured reservoirs. Fuel.

[33] Aqcheli F., Baghban Salehi M., Taghikhani V., Pahlevani H. (2021). Synthesis of a custom-made suspension of preformed particle gel with improved strength properties and its application in the enhancement of oil recovery in a micromodel scale. J. Pet. Sci. Eng..

[34] Esfahlan M.S., Khodapanah E., Tabatabaei-Nezhad S.A. (2021). Comprehensive review on the research and field application of preformed particle gel conformance control technology. J. Pet. Sci. Eng..

[35] Gao J., Lu X., He X., Liu J., Zheng K., Cao W., Cui T., Sun H. (2022). Effect evaluation and action mechanism analysis of “profile control + plugging removal” after chemical flooding. Gels.

[36] Rezaeiakmal F., Parsaei R. (2021). Visualization study of polymer enhanced foam (PEF) flooding for recovery of waterflood residual oil: Effect of cross flow. J. Pet. Sci. Eng..

